# Towards a microfluidic H295R steroidogenesis assay—biocompatibility study and steroid detection on a thiol-ene-based chip

**DOI:** 10.1007/s00216-023-04816-2

**Published:** 2023-07-13

**Authors:** Caroline Despicht, Cecilie H. Munkboel, Hua Nee Chou, Peter Ertl, Mario Rothbauer, Jörg P. Kutter, Bjarne Styrishave, Andreas Kretschmann

**Affiliations:** 1 Toxicology and Drug Metabolism Group, Department of Pharmacy, Faculty of Health and Medical Sciences, University of Copenhagen, 2100 Copenhagen OE, Denmark; 2 Institute of Applied Synthetic Chemistry, Institute of Chemical Technologies and Analytics, Faculty of Technical Chemistry, Vienna University of Technology, Getreidemarkt 9, 1060 Vienna, Austria; 3 Karl Chiari Lab for Orthopaedic Biology, Department of Orthopedics and Trauma Surgery, Medical University of Vienna, Währinger Gürtel 18-22, 1090 Vienna, Austria; 4 Microscale Analytical Systems, Department of Pharmacy, Faculty of Health and Medical Sciences, Univeristy of Copenhagen, Copenhagen, OE Denmark

**Keywords:** Thiol-ene, PDMS, Biocompatibility, Microfluidics, Steroidogenesis, Cell-based assay

## Abstract

The development of cell-based microfluidic assays offers exciting new opportunities in toxicity testing, allowing for integration of new functionalities, automation, and high throughput in comparison to traditional well-plate assays. As endocrine disruption caused by environmental chemicals and pharmaceuticals represents a growing global health burden, the purpose of the current study was to contribute towards the miniaturization of the H295R steroidogenesis assay, from the well-plate to the microfluidic format. Microfluidic chip fabrication with the established well-plate material polystyrene (PS) is expensive and complicated; PDMS and thiol-ene were therefore tested as potential chip materials for microfluidic H295R cell culture, and evaluated in terms of cell attachment, cell viability, and steroid synthesis in the absence and presence of collagen surface modification. Additionally, spike-recovery experiments were performed, to investigate potential steroid adsorption to chip materials. Cell aggregation with poor steroid recoveries was observed for PDMS, while cells formed monolayer cultures on the thiol-ene chip material, with cell viability and steroid synthesis comparable to cells grown on a PS surface. As thiol-ene overall displayed more favorable properties for H295R cell culture, a microfluidic chip design and corresponding cell seeding procedure were successfully developed, achieving repeatable and uniform cell distribution in microfluidic channels. Finally, H295R perfusion culture on thiol-ene chips was investigated at different flow rates (20, 10, and 2.5 µL/min), and 13 steroids were detected in eluting cell medium over 48 h at the lowest flow rate. The presented work and results pave the way for a time-resolved microfluidic H295R steroidogenesis assay.

**Graphical abstract:**

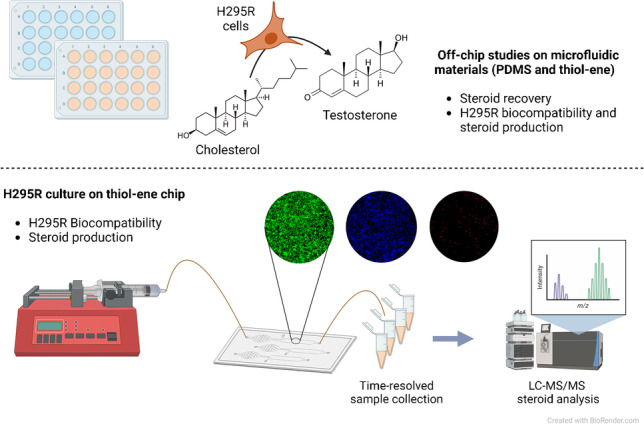

**Supplementary Information:**

The online version contains supplementary material available at 10.1007/s00216-023-04816-2.

## Introduction

Several groups of chemicals disrupt the endocrine system in humans unintentionally and are suspected to contribute to the worldwide increasing incidences of endocrine-related diseases like cancer, diabetes, obesity, and infertility [1–3]. In vitro studies performed with the human adrenocortical carcinoma cell line H295R (OECD standardized H295R steroidogenesis assay [4]) have demonstrated that several classes of drugs such as antidepressants, benzodiazepines, statins, azole antifungal drugs, and mild analgesics disrupt the synthesis of steroid hormones, which control the complex physiological processes associated with growth, reproduction, and pregnancy [5–11]. A new guidance for the pharmaceutical industry published in 2015 by the US Food and Drug Administration recommends the pre-clinical testing of drug candidates for unintended endocrine-disrupting effects [12]. This highlights the need for fast and cost-effective high-throughput in vitro assays. Existing assays like the OECD standardized H295R steroidogenesis assay [4] are quite efficient in terms of throughput, but are still time- and labor-intensive and currently not compatible with large-scale kinetic screening. A potential solution to this problem is miniaturizing existing assays using microfluidic approaches to develop live-cell micro assays. These assays operate with volumes in the microliter to nanoliter range, require reduced amounts of reagents and cells, and can integrate experimental steps like dosing, cell culturing, and drug exposure in a single microchip, making them extremely time- and cost-efficient [13–16]. Furthermore, microfluidic perfusion cell cultures offer the distinct advantages of both providing a more dynamic and in vivo like environment to human cell lines and allowing for higher time resolution in sample collection and monitoring of drug effects. For instance, a lab-on-a-chip device for the time-resolved monitoring of cytotoxic effects of nanoparticles on human lung adenocarcinoma cells under varying flow conditions was developed by Rothbauer et al. [17]. Other examples include spatiotemporal assessment of HepG2 cells in a PMMA microchip [18], and time-dependent cytotoxicity testing of anti-cancer drugs on HeLA [19] and primary human kidney cells [20]. These represent radical advancements compared to traditional in vitro cell toxicity tests with static exposure and single time point effect monitoring. However, transitioning from static well-plate systems to microfluidic devices requires operational adjustments, to sustain cells in a hydrodynamic environment and ensure nutrient delivery at reduced reagent volumes, while also limiting shear stress and excessive dilution of analytes. Furthermore, it requires moving away from standard cell culture materials like polystyrene (PS), to materials which can easily and inexpensively be prototyped and fabricated, while retaining biocompatibility and high analyte recovery [14]. Out of a variety of potentially suitable contenders (e.g., glass, PMMA, PC, and COC amongst others), PDMS has long been the gold standard in academic settings due to ease of fabrication and favorable properties such as optical transparency, gas permeability, and biocompatibility [14, 21, 22]. However, substantial drawbacks of PDMS, such as small molecule adsorption and swelling with organic solvents, have recently put novel thiol-ene polymers into the spotlight [23]. Thiol-enes have shown good biocompatibility with fibroblasts (NIH-3T3), mesenchymal stem cells (adMSCs), and epithelial (H441 and Caco-2) cells [23–25]. Casavant et al. successfully cultured H295A in PDMS microchannels [26]; however, to the best of our knowledge, microfluidic H295R cell culture has not previously been attempted. The aim of this present study was to investigate the biocompatibility of PDMS and thiol-ene for H295R cells with respect to cell morphology, cell viability, and steroid production. Furthermore, steroid absorption to chip materials was investigated via spike-recovery experiments. To allow direct comparison to the standard material (PS), these experiments were conducted in a 24-well plate format. Based on our findings, a chip design, cell seeding method, and microfluidic set-up on thiol-ene chip is proposed for the development of a miniaturized and optimized H295R steroidogenesis assay.

## Materials and methods

### Reagents and chemicals

Androstenedione (AN), pregnenolone (PREG), progesterone (PROG), dehydroepiandrosterone (DHEA), testosterone (TS), dihydrotestosterone (DHT), estrone (E1), 17β-estradiol (βE2), aldosterone (ALDO), cortisol (COR), corticosterone (COS),17α-hydroxyprogesterone (17-OHPROG), 17α-hydroxypregnenolone (17-OHPREG), 11-deoxycorticosterone (11-deoxyCOS), 11-deoxycortisol (11-deoxyCOR), and cortisone (CORNE) were purchased from Sigma-Aldrich (Glostrup, Denmark). Deuterated analogues were applied as internal standards (IS); d_7_-androstenedione (ANd_7_), d_4_-estrone (E1d_4_), d_5_-17β-estradiol (βE2d_5_), d_8_-corticosterone (COSd_8_), and d_8_-11-deoxycorticosterone (11-deoxyCOSd_8_) were obtained from CDN isotopes (Pointe-Claire, QC, Canada), while d_9_-progesterone (PROGd_9_), d_3_-testosterone (TSd_3_) and d_3_-dihydrotestosterone (DHTd_3_), d_7_-aldosterone (ALDOd_7_), and d_4_-cortisol (CORd_4_) were purchased from TRC. d_5_-11-deoxycortisol (11-deoxyCORd_5_) and d_6_-dehydroepiandrosterone (DHEAd_6_) were obtained from Sigma-Aldrich. Acetonitrile, methanol, ethanol, and sodium hydroxide (32%) were purchased from VWR chemicals (Søborg, Denmark). Formic acid 98–100% was purchased from Merck (Merck KGaA, Darmstadt, Germany). All H_2_O used was ultrapure water produced by a Milli-Q system (Millipak 40). The H295R human adrenocortical carcinoma cell line was obtained from American Type Culture Collection (ATCC, #CRL-2128, Manassas, VA, USA). Cells were cultured in 75 cm^2^ (or 25 cm^2^) flasks from Sigma-Aldrich (Brøndby, Denmark), 0.5% trypsin-EDTA and Dulbecco’s modified Eagle’s medium and Ham’s F-12 Nutrient mixture (DMEM/F12) medium (GibcoBRL Life Technologies, Nærum, Denmark) supplemented with 10 mL/L of ITS + premix and 25 mL/L Nu-serum from BD Bioscience (Brøndby, Denmark). Phosphate-buffered saline (PBS) tablets was purchased from OXOID (Hampshire, UK). Collagen Type IV, resazurin sodium salt, Live/Dead Assay Cell Double Staining Kit, and bisBenzimide H 33342 trihydrochloride (Hoechst 33342) were purchased from Sigma-Aldrich. PMMA plates were acquired from Nordic Plast A/S (Randers, Denmark). The Sylgard 184-poly(dimethylsiloxane) (PDMS) elastomer kit was purchased from Dow Corning (Midland, MI, USA). Pentaerythritol-tetrakis (3-mercapto propionate) (“tetrathiol”), triallyl-1,3,5-triazine-2,4,6(1H,3H,5H)-trione (“triallyl”) was obtained from Sigma-Aldrich (Brøndby, Denmark). Tygon tubing (ID 0.51 mm; OD 0.85 mm) was purchased from IDEX Health & Science GmbH (Wertheim, Germany) and PEEK (ID 0.381 mm) tubing was obtained from Mikrolab (Højbjerg, Denmark).

### H295R cell culture

H295R cells were cultured in accordance with the T456 OECD guideline [4], with minor modifications as described previously [7] and used for experiments between passages 4 and 13. In brief, H295R cells were cultured in T25 flasks with 10 mL growth medium at 37 °C in a 5% CO_2_ atmosphere. The growth medium consisted of DMEM/F12, Nu-serum (a low protein alternative to fetal bovine serum), and ITS premix. The cell medium was changed every 2nd to 3rd day by washing cells with 5 mL PBS and providing 5 mL fresh growth medium. Cells were split approximately once a week when confluency reached 75–90%. Cells were washed with 5 mL PBS and trypsinized with 1 mL 0.5% Trypsin-EDTA in PBS (1:10) for 3–5 min at 37 °C. Trypsinization was stopped with 3 mL growth medium, and the cells were equally split into 3 new T25 flasks, filled up to 10 mL with growth medium. As Nu-serum contains steroid hormones, it was only used in culture flasks but omitted from cell medium in experiments. For seeding, cells were trypsinized and diluted to the desired concentration (3 × 10^5^–5 × 10^6^ cells/mL, depending on the experiment).

### Coating of 24-well-plates

For biocompatibility and steroid spike-recovery experiments, well bottoms of standard polystyrene (PS) 24-well plates were covered with either PDMS or thiol-ene. PDMS mixture was prepared by thoroughly mixing PDMS base (silicone elastomer) with curing agent (silicone elastomer curing agent) in a 10:1 ratio and degassed until the mixture was clear and free of bubbles. Three hundred microliters of the mixture was pipetted into corresponding wells and the well-plate was baked in an oven at 80 °C for 2 h. PDMS-coated wells were treated with an oxygen plasma activator (Atto plasma chamber, Diener electronic GmbH & Co. KG, Ebhausen, Germany) for 1 min. The thiol-ene mixture was prepared, by mixing “thiol” (pentaerythritol tetrakis or PTMP) and “ene” (1,2,5-triallyl-1,3,5-triazine-2,4,6-(1H,3H,5H)-trione or TTT) monomers in a stoichiometric ratio, with respect to the functional groups (3 mol PTMP to 4 mol TTT). Three hundred microliters of the mixture was pipetted into corresponding wells and cured under UV-light (Dymax EC 5000 Series UV curing flood lamp, Dymax Corp, Torrington, CT, USA) for 5 min, then baked in an oven at 80 °C for 72 h. Prior to collagen coating, thiol-ene-coated wells were washed with 1 mL 70% ethanol for 10 min, followed by 10-min evaporation time after removal. The plate was then washed with 1 mL 1 M NaOH for 10 min, and 1 mL PBS for another 10 min. Where collagen coating was required, 1 mL of a 0.01 mg/mL human placenta collagen type IV solution was added to each well and incubated at 37 °C in a 5% CO_2_ atmosphere for 2 h, after which the remaining liquid was removed.

### Thiol-ene chip design and fabrication

**Fig. 1:**
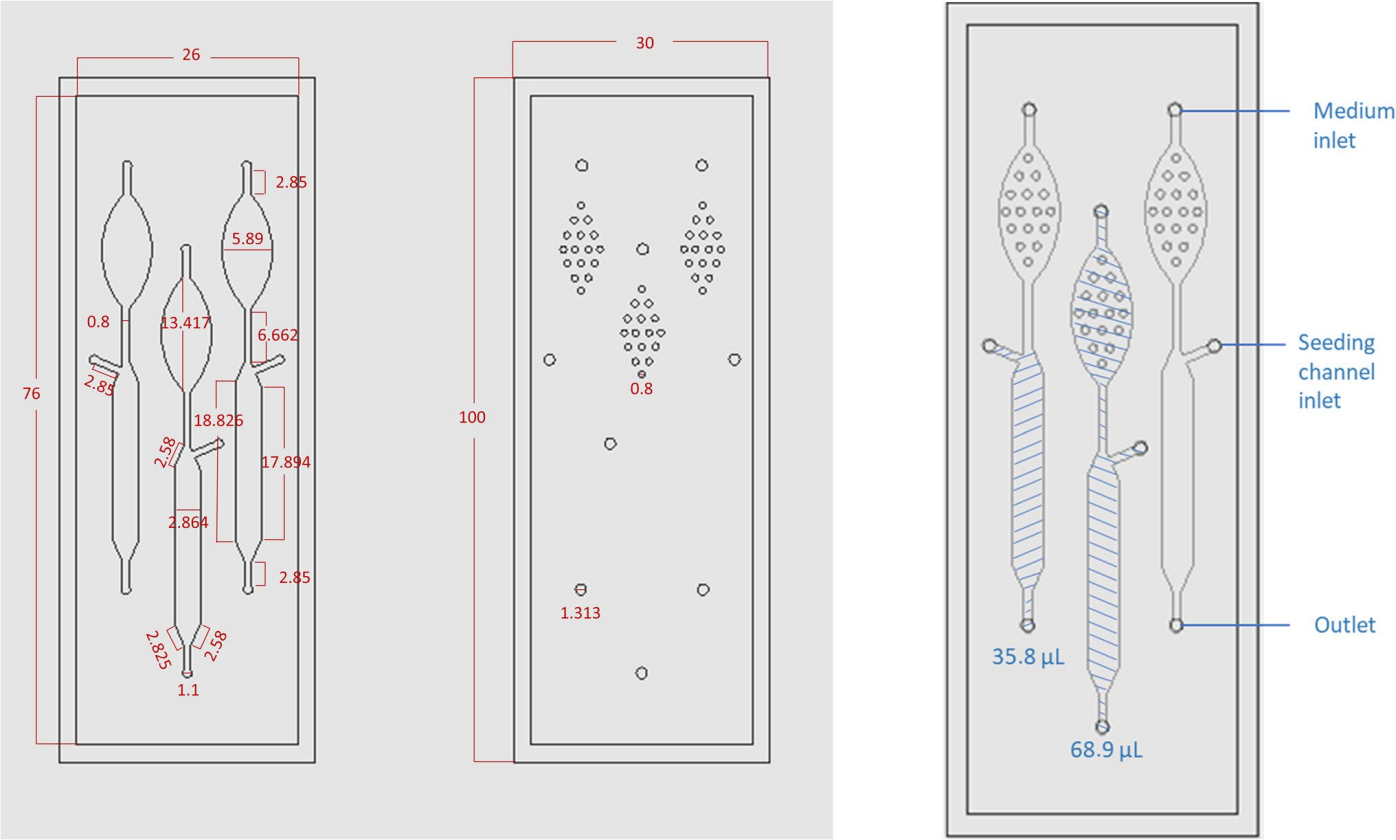
Three-channel chip design. On the left, the drawing of both chip halves is shown with dimensions in millimeters. Channel depth: 500 μm. Depth of bubble trap microwells: 250 μm. Depth of inlet holes: 1 mm. On the right, assembled chip halves with main chamber volume and total channel volume. Total chip thickness: 2 mm

Microfluidic chips were fabricated via a double-molding process, previously described by Sikanen et al. [27]. Chip structures (Fig. 1) were drawn using Autodesk Inventor Professional software (Autodesk Inc., San Francisco, CA, USA) and the PMMA master mold was obtained by high-precision milling (Minitech 3, Minitech Machinery Corp., Norcross, GA, USA). The second mold was made by pouring PDMS mixture into the PMMA master mold and baking for 2 h at 80 °C. The finished product then served as the final mold for thiol-ene chip fabrication: thiol-ene mixture (3 mol PTMP to 4 mol TTT) was filled into the PDMS mold, which was covered with a PDMS lid of the same size and placed in a UV-light source for curing (90 mW/cm^2^ at a 365-nm wavelength). The chip halves were cured between 12 and 25 s on each side. The chip halves were assembled manually and then cured under UV-light for 5 min on each side, to allow for bonding between the layers. The final product was baked at 120° C for 72 h. After cooling, 1–2-cm-long pieces of Tygon tubing were attached to the chip, using epoxy adhesive glue (EA9492 LI, LOCTITE, Henkel & Cie, Düsseldorf, Germany).

### Biocompatibility experiments

The suitability of thiol-ene and PDMS as microfluidic chip materials was investigated by growing H295R cells in 24-well plates, with well bottoms coated with PDMS and thiol-ene, respectively. Pristine PS wells served as control. Additionally, some of the wells were coated with collagen IV, to investigate whether extracellular matrix (ECM) coating favors cell growth. The “PDMS plate” consisted of uncoated PDMS, PDMS coated with collagen, and uncoated PS (control), each in 6 replicates. Accordingly, the “thiol-ene plate” consisted of uncoated thiol-ene, collagen-coated thiol-ene, and uncoated PS (control), each in 6 replicates. Well-plate arrangement is depicted in Fig. S1*.* Cells were seeded in plates with a cell density of 3 × 10^5^ in each well. Cell growth and morphology were continuously monitored over 72 h, by capturing live-images with a standard microscope. Endpoint cumulated steroid production was measured after 24 h (corresponding to the acclimatization time point in the OECD guideline, prior to drug exposure [4]). Plates were incubated for another 48 h and final cell viability was measured by Alamar blue assay after 72 h, as described by Munkboel et al. [6]. For statistical analysis, cell viability and steroid production values on pristine PS wells served as control and were normalized to 100%. Values obtained for PDMS and thiol-ene were expressed as a percentage of PS control, and analyzed by means of two-way ANOVA, followed by post hoc Dunnett’s test. The effect of collagen coating on cell viability for each material was additionally analyzed using an unpaired *t*-test.

### Steroid spike-recovery experiments

Two 24-well plate bottoms were covered with PDMS and thiol-ene, respectively, arranged as shown in Fig. S2. The “PDMS plate” consisted of 12 wells with uncoated PDMS, and 12 wells with collagen-coated PDMS. Accordingly, the “thiol-ene plate” consisted of 12 wells with uncoated thiol-ene, and 12 wells with collagen-coated thiol-ene. On an extra plate, six pristine PS wells served as control. A 14-steroid mix, with each steroid at a concentration of 10 ng/μL in methanol, was prepared. This mix was further diluted to 1 ng/mL with experimental medium, yielding a final MeOH concentration of 0.01%. Of this mix, 1 mL was added to each well and incubated at 37 °C in a 5% CO_2_ atmosphere for 48 h. Cell medium was subsequently transferred to Eppendorf tubes, to which 50 μL IS were added. 1 ng/mL steroid mix in cell medium served as reference solution. All samples were frozen to  − 20 °C until clean-up for LC-MS/MS analysis. For statistical analysis, the amount of steroids quantified in cell medium from wells with PS, PDMS, or thiol-ene was expressed as percentage of reference solution. Steroid recoveries from chip materials were compared to standard material PS by means of 2-way ANOVA, followed by post hoc Dunnett’s test.

### Microfluidic set-up and cell seeding

The experimental set-up for the microfluidic chip consisted of a syringe pump (KD Scientific Inc, Holliston, MA, USA.) holding a plastic syringe with cell medium, a thiol-ene microchip, and a fraction collector (Gilson, Middleton, WI, USA) connected by PEEK tubes. The microchip was positioned on a plastic plate with a transparent glass window for optical inspection under an inverted microscope. An in-house made heating station was used to keep the temperature stable at 37 °C for the syringe pump and the microchip. One day before cell seeding, the microchip was disinfected with 70% ethanol and rinsed with sterile PBS, each for 15 min and at a flow rate of 20 µL/min. Subsequently, 0.01 mg/mL collagen IV was introduced to coat the chip for cell attachment and left in the cell culture incubator overnight. To remove the extra collagen before use, the chip was rinsed with cell medium for 20 min at a flow rate of 10 µL/min. Cells were then seeded at a 5·10^6^ cells/mL density, by inserting a pipette tip containing 200-μL cell suspension into the seeding channel inlet. The cell suspension was sucked onto the chip by gently pulling on a syringe connected to the channel outlet via a PEEK tubing. The chip was then moved to the cell culture incubator for 2 h to allow cell attachment and subsequently connected to flow. In the current set-up, the chip’s surface area to volume ratio (SA/V) was 4 mm^2^/µL. With a culture chamber volume of 35.8 µL and initial seeding conc. of approx. 1.8·10^5^ cells/channel, this corresponds to a cell-to-volume ratio of 0.2 nL/cell (which further decreases as cells attach and grow over 48 h).

### Assessment of cell seeding uniformity and repeatability

Cell seeding uniformity and repeatability were assessed by seeding a 2·10^6^ cells/mL suspension into 2 channels. After seeding, the chip was moved to an incubator (37 °C, 5% CO_2_) for 2 h, to allow for cell attachment before counting. The chip was then inspected under a Nikon Eclipse Ti2-U inverted microscope (Nikon Corporation, Tokyo, Japan) and cells were counted manually in 9 pre-defined squares (1 mm × 1.2 mm) within a channel. To allow for comparison in cell distribution between seeding events, cell counts for each position were normalized to their relative deviation from the channel mean: $\mathrm{Relative\:deviation\:from\:mean }\left(\mathrm{\%}\right)=\frac{\mathrm{Cell\:count\:per\:square}\:-\:\mathrm{Mean\:cell\:count\:per\:channel}}{\mathrm{Mean\:cell\:count\:per\:channel }} \times 100\%$

The experiment was conducted in triplicate, using a different cell suspension on different days. Relative deviations from each replicate were pooled and plotted against their channel position, to determine whether some chip areas consistently exhibit higher cell densities than others. A one-way ANOVA was conducted to determine whether systematic deviations in cell density occur in certain channel regions. Additionally, the horizontal and vertical distribution of cells (along the length and width of the channel, respectively) was examined. For horizontal analysis, positions were pooled into front (1,2,3), middle (4,5,6), and end (7,8,9) positions of the channel as shown in Fig. 4A. For vertical analysis, positions were pooled into top (1,4,7), middle (2,5,8), and bottom (3,6,9), with the “top” positions located on the same side as the seeding channel. Pooled relative cell counts were plotted against their position on the chip, and a one-way ANOVA was conducted to determine significant differences in cell distribution in specific chip areas.

To determine whether cell numbers between channels vary significantly between seeding attempts (repeatability), cell count data was pooled and plotted against different days for visualization. A two-way ANOVA was conducted to determine whether cell counts per channel were significantly different when using the same or different cell suspensions (intra- and inter-day repeatability).

### H295R cells on-chip experiments

Cells were seeded on-chip as described above, at a 5·10^6^ cells/mL density. In a first experiment, cell morphology and attachment at a 20 µL/min and 10 µL/min flow rate were monitored over 48 h using an inverted microscope. Cell distribution and viability on-chip after 24 h of 10 µL/min flow rate was assessed using fluorescent cell stains. For this purpose, Hoechst 33342 (10 µg/mL) and live/dead assay dyes (2 μM calcein-AM and 1.5 μM propidium iodide) were injected at 5 µL/min flow rate over 30 min. The corresponding filter blocks for TRITC (550/570 nm), FITC (500/530 nm), and DAPI (360/450 nm) were used for imaging. In a second experiment, cellular steroid production was measured at flow rates of 10 and 2.5 µL/min. For this purpose, eluting medium was collected in 6-h fractions, over a 48-h period. The experiments were conducted in triplicate at each flow rate. Samples were stored in  − 20 ℃ until sample clean-up.

### Sample preparation and steroid analysis

Samples from well-plate experiments were extracted using the double protein precipitation method developed by Weisser et al. [28]. As cell medium samples from the chip were smaller in volume (450 μL instead of 950 μL), clean-up was optimized to increase steroid recovery and involved a single-step protein precipitation with acetonitrile in a ratio of 1:3 (v/v). One thousand four hundred fifty microliters of cold acetonitrile and 50 μL of 0.1 ng/μL IS (Steroid-Dx mix containing deuterated steroid analogues used as internal standards) were added to each sample tube and mixed thoroughly. The mixture was then centrifuged at 1800 rcf for 10 min. Supernatants were transferred to HPLC vials and evaporated down to approximately 0.5 mL under at gentle stream of nitrogen at 60 °C. Finally, the vials were filled up to 1 mL with MeOH:H_2_O (20:80, v/v) and stored at  − 20 °C until quantification. The fully validated LC-MS/MS method by Weisser et al. [28] was employed for chromatographic separation and simultaneous determination of 16 steroids, with minor modifications. In brief, a binary 1290 Agilent Infinity Series system and a quaternary 1100 Agilent HPLC pump were combined to conduct online SPE clean-up, chromatographic separation, and detection. The process involved using a C18 enrichment column (3.9 × 20 mm, 10 μm particle size), transferring to a C18 guard column (2.1 mm), and separating on a C18 analytical column (75 × 2.1 mm, 2.5 μm particle size). Each sample (100 μL) was injected through a 0.3-μm in-line filter onto the enrichment column and flushed with a solvent composition of H_2_O/MeOH/formic acid (80:20:0.1, v/v/v) for 2 min to eliminate salts and proteins while retaining analytes. The chromatographic separation involved a gradient starting with 80% mobile phase A (H_2_O with 0.1% formic acid) and 20% mobile phase B (pure MeOH). Over 14 min, the proportion of mobile phase B was gradually increased to 98% at a flow rate of 0.5 mL/min. Steroids were detected using an AB SCIEX 4500 QTRAP mass spectrometer equipped with an atmospheric pressure chemical ionization (APCI) source. The chromatographic data was analyzed, and peak areas were corrected manually where necessary using Analyst and MultiQuant 3.0 software (AB SCIEX, Framingham, MA, USA). Quantification of steroids was performed with a calibration curve ranging from 0.01 to 200 ng/mL and data was further processed using Microsoft Office Excel (Microsoft Corporation, Redmond WA, USA) and GraphPad Prism v. 9.04. (GraphPad Software, San Diego, CA, USA). Lower limit of detection (LLOD) and lower limit of quantification (LLOQ) were determined as described by Weisser et al. [28].

## Results

### Biocompatibility of biochip materials

**Fig. 2:**
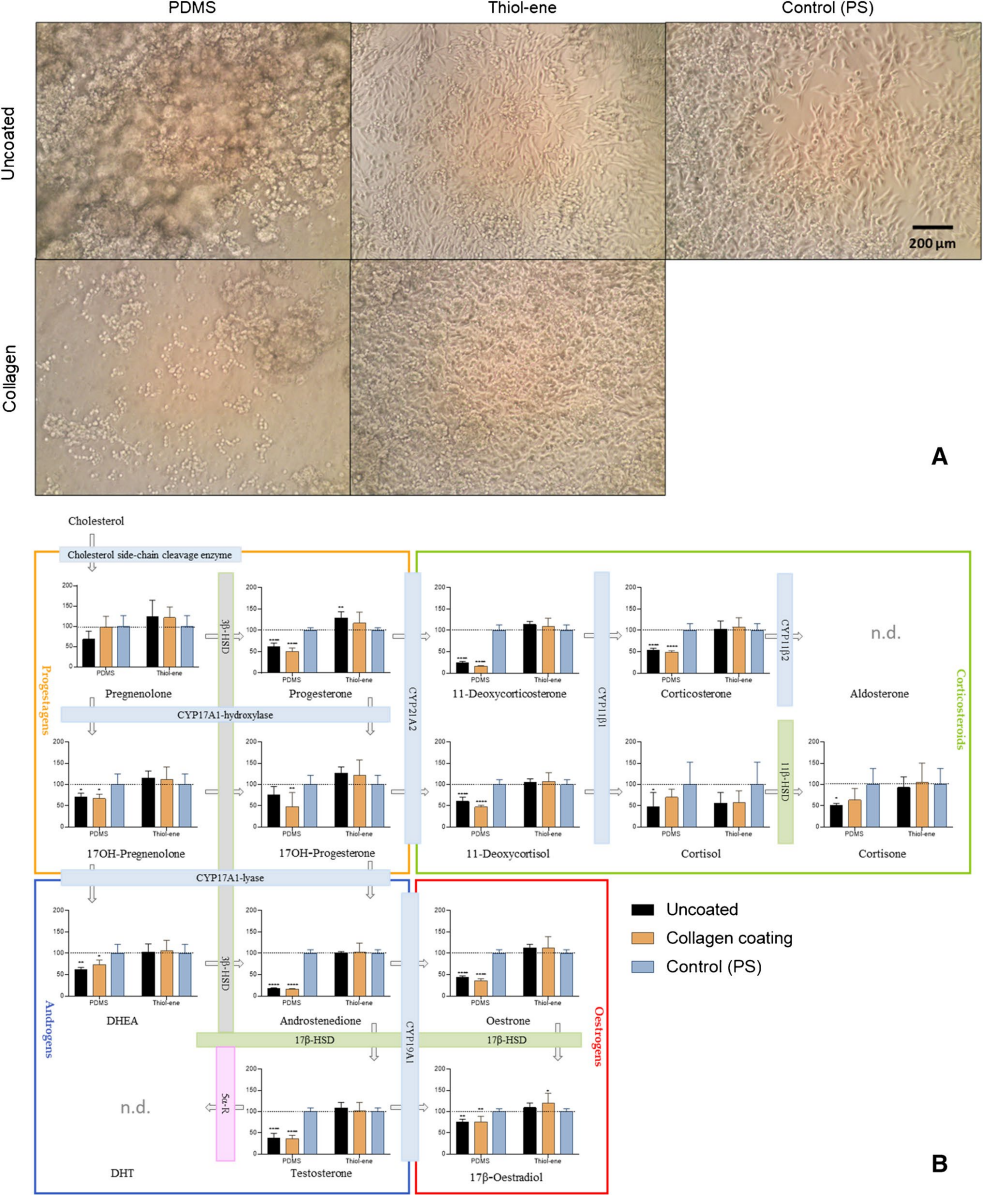
H295R cell biocompatibility on PDMS and thiol-ene. **A** Microscope images of H295R cell attachment taken in the center of standard wells covered with PDMS and thiol-ene discs 24 h after seeding, with and without collagen coating. Uncoated (PS = polystyrene) wells served as controls on each well-plate. **B** Steroid levels in cell medium collected after 24-h incubation of H295R cells on PDMS and thiol-ene discs, with and without collagen coating. Steroid levels are expressed as percentage of polystyrene control (PS = 100%; horizontal line). Values represent mean ± SD (*n* = 5–6 technical replicates). Asterisks indicate significant difference from control (one-way ANOVA, post hoc Dunnett’s test). Significance levels: *p ≤ 0.05, **p ≤ 0.01, ***p ≤ 0.001, and ****p ≤ 0.0001. n.d., not determined. Blue bars indicate cytochrome P-540 enzymes; grey and green bars indicate hydroxysteroid dehydrogenase enzymes; pink bar indicates 5α-reductase, depicting enzymes involved in steroidogenesis pathway [29]

Biocompatibility of potential microchip materials (PDMS and thiol-ene) and ECM coating agent collagen with H295R cells was assessed by means of cell attachment, cell viability, and steroid secretion into the cell medium. Figure 2A shows H295R cell attachment on PDMS and thiol-ene discs in the well-plate format, compared to a standard PS control well, after 24 h of acclimatization. On PDMS, cell distribution was highly uneven: some areas showed cloudy areas of cell aggregations while neighboring stretches only had few, round, and luminous cells, indicating failure to attach and/or cell death. Clumping was observed both on pristine and collagen-coated PDMS; however, collagen generally improved cell proliferation and viability (Table S1). Accordingly, steroid concentrations measured in PDMS wells were significantly lower compared to PS control (Fig. 2B); however, it should be noted that aside from biological activity, adsorption and/or sequestration of steroids into PDMS (addressed in the next section) could be a contributing factor. On uncoated thiol-ene, cells displayed the characteristic elongated shape of a 2D-monolayer and cell confluence (~ 80 to 100%) was comparable to PS, with collagen coating further promoting cell proliferation (confluence  > 100%, Fig. 2A). Interestingly, cell viability was up to 20% higher on both native and collagen-coated thiol-ene, compared to standard well-plate material PS (Table S1). Accordingly, measured steroid levels were comparable or increased on thiol-ene discs in relation to PS (Fig. 2B), with the exception for cortisol, which was slightly lower on thiol-ene. However, due to high variations in cellular cortisol secretions, this difference is not statistically significant. Numerical values for steroid concentrations measured in cell medium are listed in Table S2. In general, the thiol-ene material gave better attachment, viability, and steroid recovery than the PDMS material. Furthermore, collagen coating had no major impact on steroid production but appeared favorable to cell proliferation on both materials. As collagen offered a slight but statistically significant improvement in cell viability on thiol-ene (Table S1), collagen was maintained in further experiments on thiol-ene chips.

### Impact of biochip material on steroid recovery

**Fig. 3:**
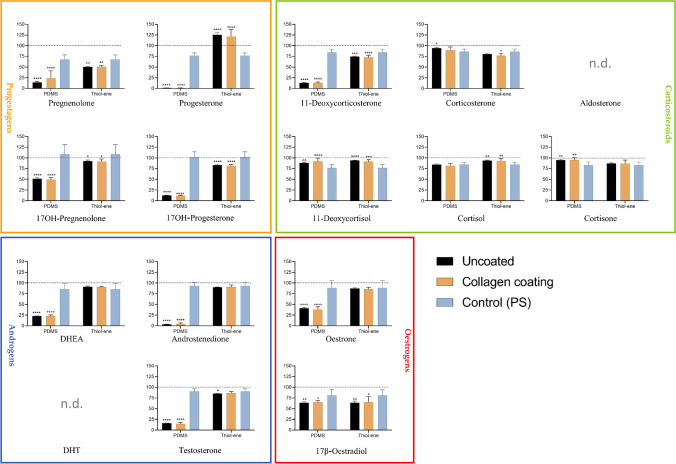
Absolute steroid recoveries (*n* = 6–12 technical replicates) of 1 ng/mL spiked cell medium collected after 24-h incubation (*y*-axis) on PDMS and thiol-ene discs, with and without collagen coating, as well as PS control (*x*-axis). Steroid levels are expressed as percentage of 1 ng/mL steroid mix reference solution spiked in cell medium (= 100%; dotted line). Symbols and legends otherwise as in Fig. 2

To ensure sensitive detection of steroids and prevent unnecessary losses on chip materials, steroid adsorption to PDMS and thiol-ene was examined in the well-plate format. In this experimental set-up, it is important to note that as only well-plate bottoms were covered with microfluidic chip materials, steroids were still in contact with walls of PS wells. The reported recoveries thereby result from a mixed effect of microfluidic chip materials and PS but give a significant insight of steroid adsorption to PDMS and thiol-ene. As observed in Fig. 3, absolute recoveries from standard well-plate material PS were  > 65% for all steroids, but rather low on PDMS, with PREG, PROG, 17-deoxy COS, DHEA, AN, and TS below 25%, and 17-OH PROG, E1, and β-E2 below 65%. Recovery from thiol-ene was comparable to the PS control for most steroids, while the few steroids with lower recovery than the control (PREG, 11-deoxy COS, COS, TS, β-E2) remained within 20% of the recovery on PS. Numerical values for the recoveries of each steroid are listed in Table S3. Surprisingly, PROG recovery from thiol-ene was increased by 122–125% compared to the spiked medium control (+ 45% compared to PS), but also prone to high variation. While we initially hypothesized collagen might act as a protective layer to adsorption, by preventing steroids from interacting with the chip material, this does not appear to be the case, as recoveries are similar with and without coating.

### Optimization of on-chip cell seeding procedures

**Fig. 4:**
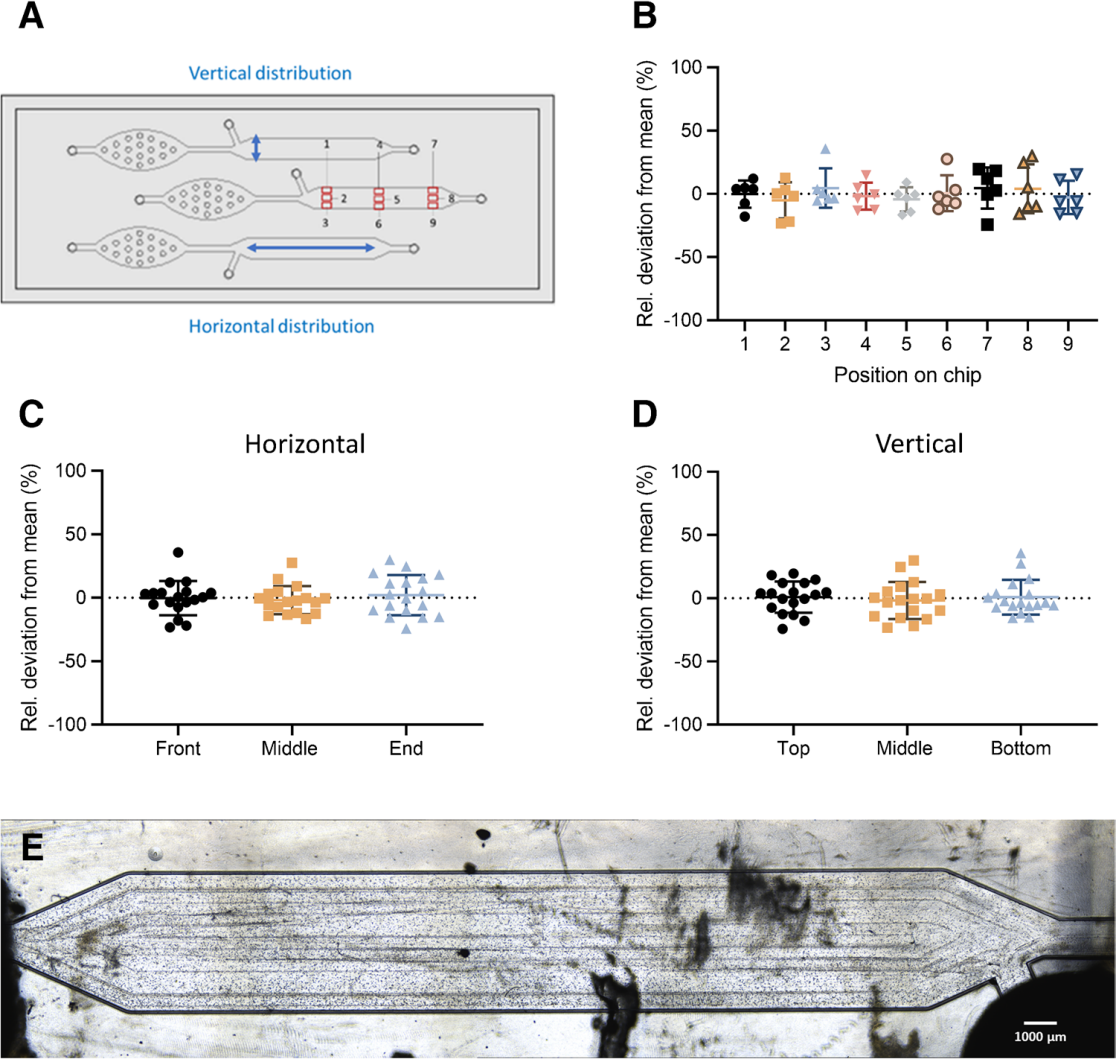
**A** Thiol-ene chip design with red squares indicating cell counting areas on chip (of 1.2 mm^2^ each) and blue arrows illustrating horizontal and vertical cell distributions. **B** Cell distribution on-chip illustrated as percentage deviation from the mean cell count per channel (*x*-axis) for each position (*y*-axis). Values are represented as mean ± SD (*n* = 6). **C** Horizontal distribution, with positions from **B** pooled into front (1,2,3), middle (4,5,6), and end (7,8,9). **D** Vertical distribution, with positions from **B** pooled into top (1,4,7), middle (2,5,8), and bottom (3,6,9). No significant differences in cell densities were observed between positions (one-way ANOVA). **E** Representative microscope image of the main channel area, taken immediately after seeding. Dark stains are scratches on the thiol-ene chip surface

As collagen-coated thiol-ene displayed favorable properties for H295R cell culture, a microfluidic chip design with corresponding cell seeding procedure was elaborated next. The proposed seeding method (via seeding inlet separated from the bubble trap) effectively directed cells into the main channel, allowing for cell counting and monitoring in a clearly defined area. A video file showing live cell seeding and distribution onto the chip is provided as electronic supplementary material Video S1. Cell counts in 9 different positions on-chip (Fig. 4A) revealed no tendency towards cell clustering in a specific channel area, neither horizontally, nor vertically (Fig. 4B–D) indicating uniform cell distribution in the channel, as seen in Fig. 4E. This was confirmed by statistical analysis (one-way ANOVA). Detailed cell seeding uniformity values are listed in Table S5. Using the same cell suspension for seeding led to comparable cell densities in both channels, while cell densities only varied by up to ± 20% with independently prepared cell suspensions. On average, cell seeding with a 2 ·10^6^ cells/mL suspension resulted in approximately 4 ·10 ^4^ cells per channel (50–60% confluence) after cells were allowed to settle for 2 h without flow. Detailed cell seeding repeatability values are shown in Table S6 and Fig. S4.

### Performance evaluation of thiol-ene biochip-based H295R cultivation

After demonstration of chip material and seeding method suitability, H295R cell performance on collagen-coated thiol-ene chips was assessed at different flow rates in terms of cell viability, cell morphology changes, and basal steroid production over 48 h.

**Fig. 5:**
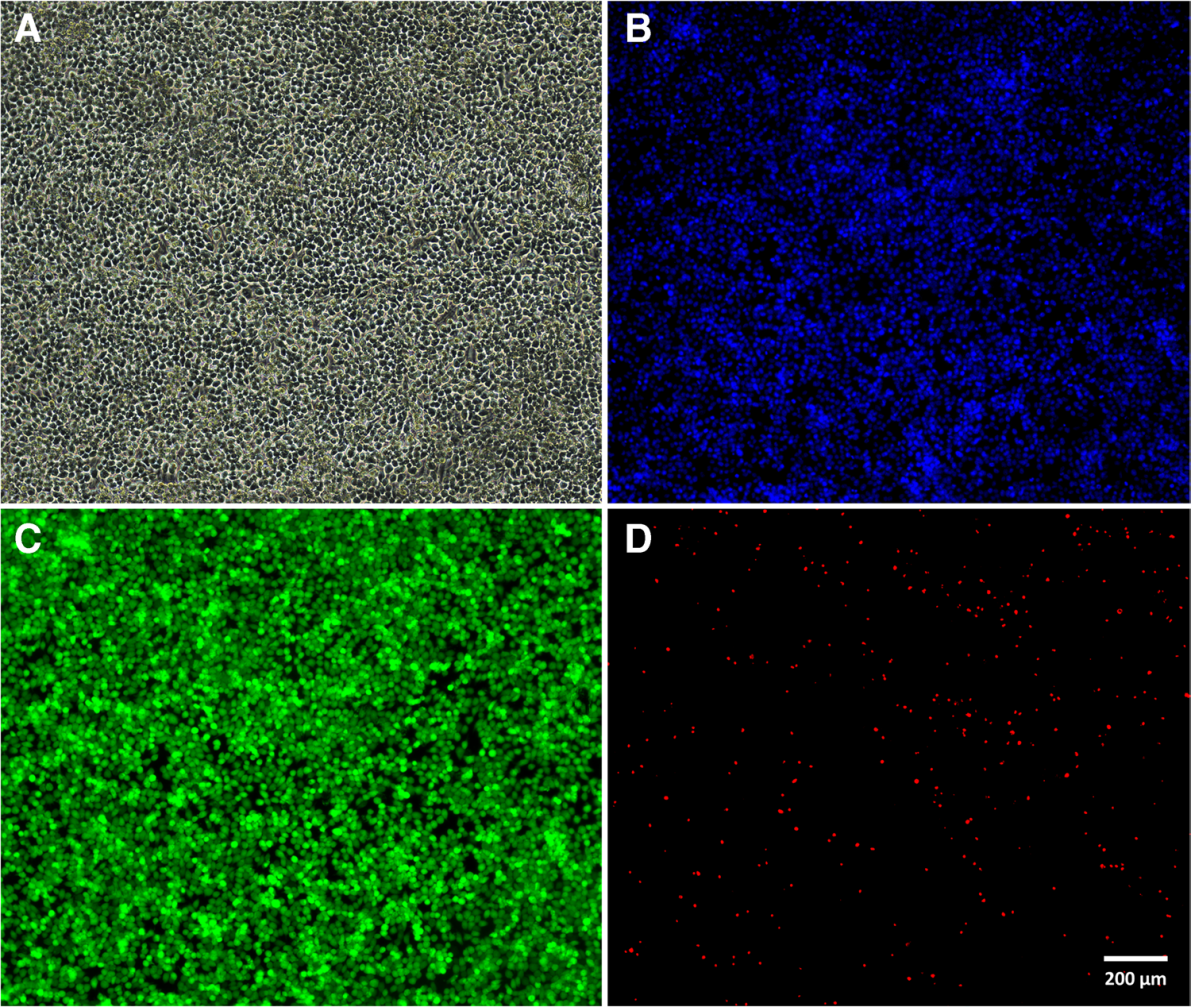
H295R cells on collagen-coated thiol-ene chip, 24 h after seeding (flow rate 10 µL/min). All pictures were taken in the same position with a standard fluorescence microscope (20 × magnification). **A** Brightfield and **B** Hoechst nuclei staining (DAPI filter). **C** Live cells (TRITC filter) and **D** dead cells (FITC filter)

**Fig. 6:**
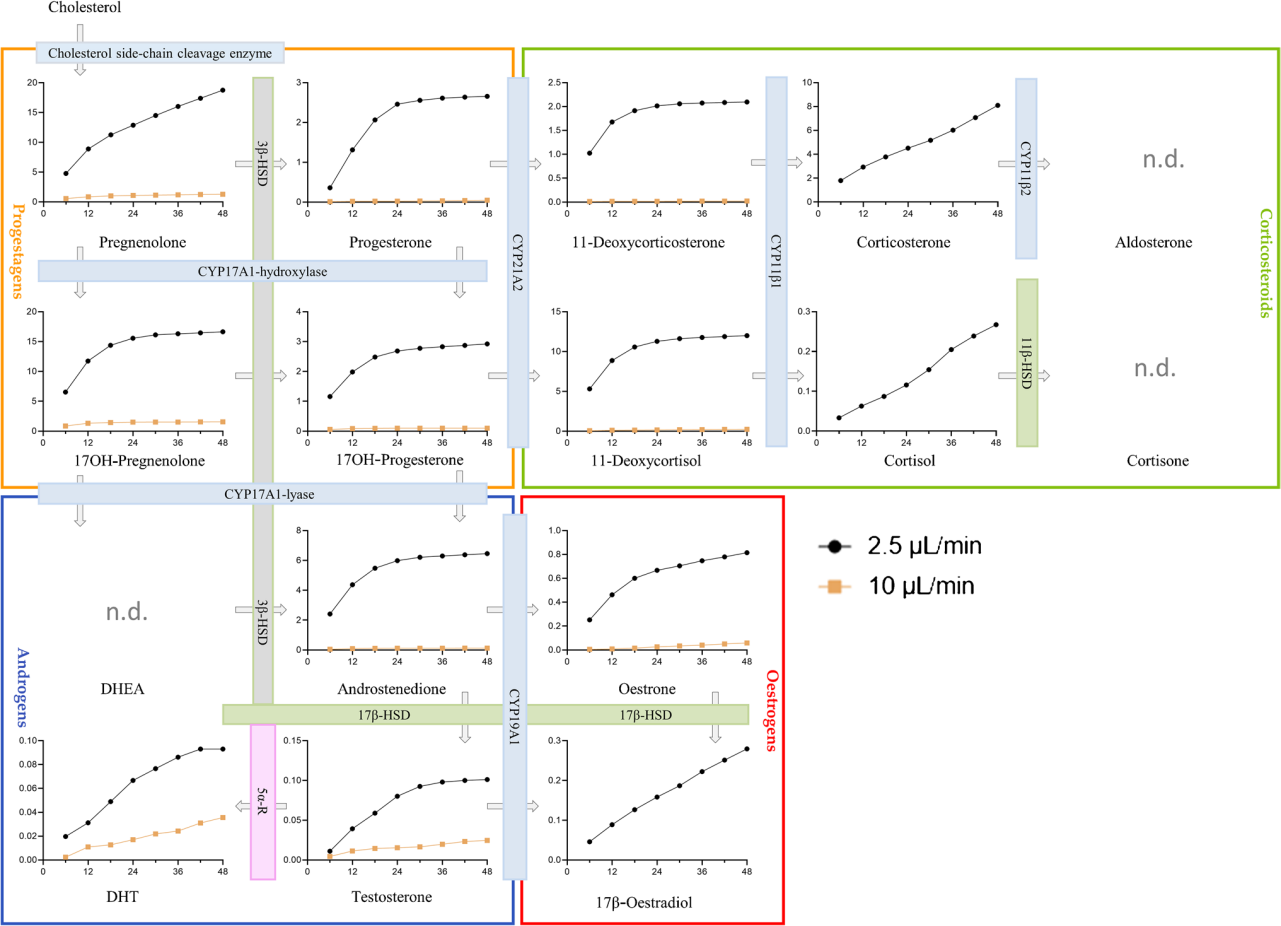
Mean cumulative H295R steroid production on thiol-ene chip (*n* = 3–4 independent experiments) in ng/mL (*y*-axis). Samples were collected in 6-h fractions over 48 h (*x*-axis). Blue bars indicate cytochrome P-540 enzymes; grey and green bars indicate hydroxysteroid dehydrogenase enzymes. Pink bar indicates 5α-reductase

For on-chip experiments, H295R cells were first left to settle for 2 h without flow, yielding a 2D-monolayer on the channel surface (confluence  ~ 80%). Applying 20 µL/min flow caused cells to be flushed out of the channel immediately, and further experiments at this flow rate were therefore discontinued. At 10 µL/min, cells retained their characteristic, dark elongated shape over 48 h (final confluence  ~ 90%). The corresponding time-resolved image series, showing initial cell attachment (without flow) and unchanged morphology under 10 µL/min flow is provided in Fig. S5. Furthermore, cell viability experiments showed no signs of cell stress on microscope images, taken 24 h after initiation of 10 µL/min flow. Figure 5A demonstrates full cell attachment to the chip surface (confluence  ~ 100%) after 24 h, while nuclei (Fig. 5B) and cytoplasm staining (Fig. 5C) revealed some cell growth beyond the monolayer, as some cells appear smaller and fainter in the image’s background. Live/dead staining showed very high cell viability (Fig. 5C, green) and low cell mortality (Fig. 5D, red), even under flow conditions. However, H295R steroid secretions in 10 µL/min samples collected in 6-h fractions appeared too diluted in to be detected at reasonable concentrations, with COR and 11-deoxy COS below the LLOQ, whereas PREG, 17-OH PROG, TS, DHT, and E1 were below the LLOD, and COS and E2 were not detected at all (Fig. 6). Lowering the flow rate to 2.5 µL/min considerably improved steroid detection, with most steroids (except TS, DHT, and E1) measured above the estimated LLOQ in the first 24 h. Here, cumulative steroid secretion patterns show an initial increase which flattens out for most steroids after about 24 h. A timeline plot of steroid synthesis is provided in Fig. S6 and a detailed table of measured steroid levels compared to LLODs and LLOQs is shown in Table S7.

## Discussion

### Chip material

PDMS has been widely adopted as a microfluidic chip material due to its (perceived) biocompatibility, low cost, and ease of fabrication. Challenges associated with PDMS are adsorption of small molecules, gas permeability, and swelling due to solvents. Moreover, PDMS is highly hydrophobic and surface treatments (e.g., UV-ozone, oxygen plasma) and/or extracellular matrix coating are usually required to render the surface more hydrophilic and suitable to mammalian cell culturing [14, 21, 23, 30–34]. However, hydrophilic surface modification is only temporary and PDMS has been shown to return to its hydrophobic state over time [35–37]. This also appears to be the case in our study, as cells aggregated in clumps and/or failed to attach on PDMS despite oxygen plasma treatment and collagen coating. In spite of this, cells were alive (67–98% cell viability), and synthesized steroids could be detected albeit in considerably lower concentrations than on standard PS. There are two possible explanations for this: firstly, uneven cell agglomerations can alter cellular activity and thereby steroid secretions. Secondly, hydrophobic steroids (such as PREG, PROG, 17-deoxy COS, TS, DHEA, and AN) are extensively adsorbed and/or absorbed by PDMS, affecting the equilibrium state in the steroidogenesis pathway, and limiting the formation of further downstream steroids such as COS, COR, and CORNE (Fig. 6). Midwoud et al. [38] previously conducted steroid spike-recovery experiments in a PDMS microchip and found AN and TS to be adsorbed to PDMS (~ 85–90% absolute recovery from chip), albeit to a much lesser extent than in our study (~ 4–16% absolute recovery in well-plate). These disparities are likely due to differences in cell types and properties, experimental set-up, and surface-to-volume ratios, but underline the importance of conducting separate recovery experiments for each newly developed microfluidic platform and cell line. In another spike-recovery study, Casavant et al. [26] recovered only 24–36% cortisol from their “suspended” PDMS microchip despite pentanol extraction, but argued this was sufficient for LC-MS/MS detection of TS, COR, COS, and 11-deoxy COS from H295A cell culture [26]. McDonald and Whitesides highlighted that PDMS’ suitability is highly dependent on its application [38] and PDMS microchips have previously been applied for cell culture and steroid analysis, despite documented retention of hydrophobic molecules [39–41]. However, Regehr et al. warned against PDMS’ direct interference in cell signaling pathways, after reporting extensive estrogen sequestration into PDMS inserts, which significantly inhibited estrogen-dependent cellular response. [42]

Unlike PDMS, thiol-enes are mildly hydrophilic, do not necessarily require surface treatment, and are naturally compatible with various tissue and cell cultures, such as fibroblasts (NIH-3T3), mesenchymal stem cells (adMSCs), and epithelial (H441 and Caco-2) cells, amongst others [23–25]. In our study, H295R cell proliferation was highly satisfactory in the well-plate experiments on both uncoated and collagen-coated thiol-ene discs (confluence  > 80% after 24 h), and cell viability surpassed PS controls (> 114%). Accordingly, measured steroid levels were similar or slightly higher compared to PS control wells, and steroid absorption by thiol-ene was negligible. However, PROG recovery from thiol-ene was inexplicably increased by 25% compared to spiked medium controls. As a surge in PROG levels was also observed in the biocompatibility study, we suggest taking a closer look at this hormone in further studies (i.e., to verify whether samples exposed to thiol-ene could be contaminated with chemicals interfering with PROG detection). A time-resolved spike-recovery study was also conducted on our microfluidic thiol-ene chip (Table S4) and showed consistently high steroid recoveries over 48 h for hydrophilic corticosteroids. Meanwhile, a drop in recovery was observed over time for the most hydrophobic steroids, such as PROG, PREG, 11-deoxy COS, AN, and DHEA. McManus et al. observed a similar time-dependent retention of PREG and DHEA in plastic vessels [43]. However, we suspect it is auxiliary equipment (such as plastic syringes and PEEK tubing), which likely retains a great proportion of steroids in our microfluidic set-up rather than the thiol-ene bulk material, as the chip itself accounted for less than 10% of steroid losses (Table S4). To our knowledge, no previous steroid adsorption studies have been reported for thiol-ene as a chip material, and our findings underline its excellent suitability as microchip material for H295R cell culture and steroid analysis.

### Chip design and cell seeding

Challenges associated with thiol-ene materials mainly lie with their gas impermeability and oxygen uptake, risking fast oxygen depletion in a closed microfluidic chip. This can be avoided by prolonged heat-treatment of the chip prior to use and decreased surface-volume ratios [44, 45]. Thus, a 500-μm channel height was chosen to ensure sufficient oxygen supply to the cells. Our microfluidic chip is of relatively simple design, with a straight channel for cell culturing, a medium inlet through which fluids and cells are introduced into the channel, and an outlet for collection of waste and medium samples. The bubble trap (upstream of the main culturing chamber) contains inverted microwells on the upper channel half, catching small bubbles in cell medium as they float to the surface. The seeding channel inlet allows selective seeding into the main culturing channel, circumventing the bubble trap (Fig. 1). Our cell seeding method only requires inexpensive and commonly available laboratory equipment (plastic syringes, micropipette tips, and PEEK tubing) and provided homogenous cell distribution within the channel. Furthermore, seeding with the same cell suspension resulted in comparable cell numbers on different chips, and varied between days by no more than 20% with independently prepared cell suspensions. Achieving uniform and repeatable cell density is essential in ensuring consistent steroid expression patterns between runs. The H295R steroidogenesis assay OECD guideline [4] therefore prescribes normalization of drug-exposed cells to solvent control (SC) wells, to account for plate-to-plate and day-to-day variabilities in seeded cell densities [4]. Our microfluidic chip design with parallel cell culture channels allows to run a “SC” next to a “drug exposure” channel, to fulfill this OECD quality criteria and account for assay-to-assay variability in the microfluidic system.

While several instances have called for the standardization of microfluidic cell-based assays, to the best of our knowledge, no concrete guidelines have emerged from the “Organ-on-Chip” community or regulatory bodies to this date. Thorough validation procedures (for commercialization) are usually carried out in the private sector, and hence not available to researchers [46]. Only very few publications have thus far assessed cell seeding uniformity and repeatability. In their attempt to generate a flow-free chemical gradient, Mosadegh et al. [47] assessed seeding uniformity purely visually, and seeding repeatability between devices was evaluated by comparing mean cell densities and standard deviations from different microchambers. Hemmingsen [48] used a similar method to ours to assess cell distribution in a microchannel, by comparing normalized cell counts in different channel areas. Cooksey et al. [49] determined within-device repeatability, by monitoring the degradation of green fluorescent protein (GFP) after treatment with a reversible ribosome inhibitor. Day-to-day reproducibility was monitored by replicating measurements in microfluidic devices prepared on different days. However, in this case, GFP decay-time was monitored as an endpoint rather than cell numbers. In a step towards standardization, Charwat et al. [50] developed a miniaturized cell analysis platform able to monitor cell numbers, attachment, and proliferation on chip via light scattering and impedance measurements, but its implementation for routine microfluidic live-cell assays would be extremely complex and costly. Our cell seeding uniformity and repeatability studies provide a simple framework in attempting to standardize cell seeding and could be applied to other microfluidic cell assays.

### H295R cells on chip

At a 2.5 μL/min flow rate, steroid secretion patterns from our perfused microfluidic culture resembled the static environment of the well-plate. For most steroids, a plateau was reached by 24 h after flow initiation despite constant medium renewal (at a 2.5 µL/min flow rate, complete medium renewal of the cell culture chamber takes approx. 28 min). Only COR, COS, and E2 production continued to increase, most likely because synthesis is delayed compared to further upstream steroids and plateau not yet achieved by 48 h. This corresponds to steroid synthesis patterns observed in the well-plate, with progestagen production reaching maximum concentrations earlier than estrogens, androgens, and corticosteroids [51–53]. The observed plateau in cumulated steroid synthesis observed can be attributed to several underlying factors. Firstly, autocrine feedback mechanisms can cause cells to lower their steroid synthesis. Additionally, contact inhibition resulting from cells reaching maximum confluence in the chip can further alter and/or impede steroidogenic processes. Finally, we cannot exclude that the low flow rate of 2.5 µL/min actually provides insufficient nutrients (i.e., oxygen), and that this effect worsens with ongoing cell proliferation. As our thiol-ene chip is gas-impermeable, future studies should investigate whether oxygen supply through flow is sufficient to satisfy cellular consumption over 48 h. However, it should be noted here that cells were purposely seeded at a high concentration to ensure detection of steroids, but that these cell numbers should be reduced for future experiments. As cell confluence is known to affect and steroid expression patterns, the OECD T456 guideline prescribes 50–60% confluence at the start of experiments, to ensure consistency in steroid expression between runs [4]. Lowering cells numbers while maintaining steroid levels at detectable concentrations could e.g. be achieved by up-concentration of samples (i.e., via solid phase extraction procedures) or a more sensitive detection system (i.e., UHPLC-MS/MS) [54]. While flow rate, chamber volume, and cell seeding concentration will still have to be finely tuned to create optimum conditions for H295R cells, 13 steroids were detectable and quantifiable from our microfluidic chip (Fig. 6). Many other research groups have previously opted for simultaneous determination of multiple steroid endpoints (compared to the sole assessment of E2 and TS, as per the current OECD guideline) as this can provide a deeper understanding of steroidogenic disruption mechanisms [5, 55, 56]. With a growing necessity for fast, inexpensive, and efficient screening of potential endocrine disruptors, the development of high-throughput H295R assays has previously been attempted. In their high-throughput H295R assay developed within the frame of the US EPA ToxCast program, Haggard et al. [55] included 11 steroids and increased assay efficiency by upscaling to 96-well plates and pre-stimulating cells with Forskolin, thereby detecting even the most weakly secreted hormones [57]. With their platform, they accurately screened 25 reference chemicals used in the OECD validation, with accuracies  > 75% for increased/decreased estrogen and testosterone production. However, Forskolin pre-stimulation can be problematic, as it significantly stimulates production of corticosteroids and estrogens, but to a much lesser extent progestagens and androgens [28]. In addition, synergistic and/or additive effects between forskolin and investigated xenobiotics might skew representations of potential drug-induced alterations in the pathway [58]. An alternative solution without “artificially induced” steroidogenesis would therefore be preferable.

Cell-based microfluidic platforms have recently emerged as novel high-throughput alternatives to traditional well-plate, but their throughput capabilities are highly dependent on set-up and experimental design, where scalability and compatibility with existing workflows still pose challenges. However, a decisive advantage of perfused microfluidic cell-based assays is the generation of multiple sample fractions from a single channel, which we believe will be a major driver for large-scale kinetic studies. In terms of endocrine disruption, time-resolved data collection gives the opportunity to explore detailed kinetics of endocrine disruption by chemicals (including potential reversibility of toxic effects) in a “higher-throughput” manner than is currently possible with traditional well-plate assays. A current limitation of the traditional H295R assay set-up is that it consists only of a single time point measurement, which can limit mechanistic understanding of steroidogenic regulation. Endogenous hormones (such as ACTH, angiotensin II, and orexin) have previously been shown to exert time-dependent effects on steroidogenic gene expression level [59–61] and measuring ED’s impact on steroidogenesis over time could thereby be of high relevance in their general toxicity assessment, as xenobiotics might have more short- or long-lasting effects on steroidogenesis. In a preliminary well-plate experiment, H295R steroid synthesis recovered almost completely, only 3 h after exposure to the known CYP inhibitor miconazole (data not shown), underlining the need for more time-resolved studies. Several computational models investigating kinetic H295R steroidogenesis profiles have recently been developed [51, 52, 62, 63]; however, all required manual sampling of cell medium from the well-plate, leaving longer gaps between collection time points. With a constant perfusion system and automated sample collection, our microfluidic set-up would facilitate continuous sampling for time-resolved toxicity studies; an option that can be further explored in future studies.

## Conclusion

In this work, we demonstrated thiol-ene’s excellent suitability as H295R cell culture material, in terms of biocompatibility and satisfactory recovery of 13 steroids. Our developed thiol-ene chip, seeding method, and microfluidic set-up are inexpensive, easy to assemble and operate with commonly available laboratory equipment, and thereby easily applicable within academia or for industrial purposes. The developed microfluidic H295R cell culture platform showed detectable steroid concentrations over 48 h at a 2.5 µL/min flow rate, thereby setting the premise for a time-resolved microfluidic H295R assay, which will enable assessment of an endocrine disruptor’s direct influence on the entire steroidogenesis pathway during and after drug exposure. Undoubtedly, further optimization and standardization of our microfluidic platform are still needed to provide a fully viable alternative to the existing well-plate assay. Steroid detection is a current limitation, as most steroids collected from a fully confluent cell culture channel are presently measured at their limit of detection with our established LC-MS/MS method (Table S7). A UHPLC-MS/MS method, with which Dong et al. achieved LLODs in the 0.05–0.10 pg/mL range [54], could be considered as an alternative. This would both ensure sensitive detection of steroid concentration changes and allow for further downsizing of microfluidic cell culture chambers, thereby fitting more parallel channels (e.g., triplicate “solvent control” and triplicate “drug exposure” channels) onto one chip for replicate measurements. Further steps to be taken include proof-of-concept studies on-chip with known steroidogenesis inducer forskolin and inhibitor prochloraz, to ensure detectable changes in steroid hormone expression. Finally, we consider it crucial to adapt the existing OECD T456 quality standards (i.e., regarding cell confluence, viability, and acceptable variability within hormone measurement systems) to the microfluidic format, to ensure method robustness and consistency between assays.

## Supplementary Information

Below is the link to the electronic supplementary material.

Supplementary file1 (DOCX 1.69 MB)
