# Supplementary material for: Towards a microfluidic H295R steroidogenesis assay—biocompatibility study and steroid detection on a thiol-ene-based chip
Source: Anal Bioanal Chem. 2023 Jul 13;415(22):5421–36. doi: 10.1007/s00216-023-04816-2 (PMC10444685; doi:10.1007/s00216-023-04816-2)
Supplement: Supplementary file 1 — Supplementary file1 (DOCX 1.69 MB) [file 216_2023_4816_MOESM1_ESM.docx]

# Electronic Supplementary Material

**Towards a microfluidic H295R steroidogenesis assay – Biocompatibility study and steroid detection on a thiol-ene based chip**

Caroline Despicht^a^, Cecilie H. Munkbøl^a^, Hua Nee Chou^a^, Peter Ertl^b^, Mario Rothbauer^b,c^, Jörg P. Kutter^a^, Bjarne Styrishave^a^, Andreas Kretschmann^a^

^a^ Department of Pharmacy, Faculty of Health and Medical Sciences, University of Copenhagen, 2100 Copenhagen OE, Denmark

^b^ Institute of Applied Synthetic Chemistry, Institute of Chemical Technologies and Analytics, Faculty of Technical Chemistry, Vienna University of Technology, Getreidemarkt 9, 1060 Vienna, Austria

^c^ Karl Chiari Lab for Orthopaedic Biology, Department of Orthopedics and Trauma Surgery, Medical University of Vienna, Währinger Gürtel 18-22, 1090 Vienna, Austria

Corresponding author e-mail: bjarne.styrishave@sund.ku.dk

## Supplementary material figures


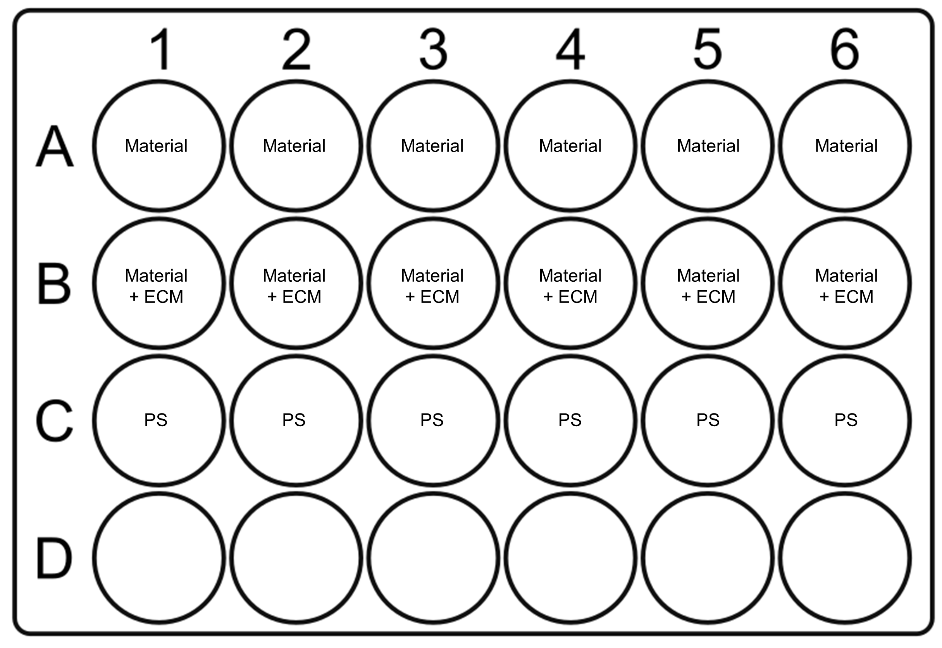


**Fig. S1** 24-well plate arrangement for testing of different chip materials (PDMS or thiol-ene) and their effect on H295R cell culture. PS: polystyrene; ECM: extracellular matrix, referring to coating agent collagen IV.


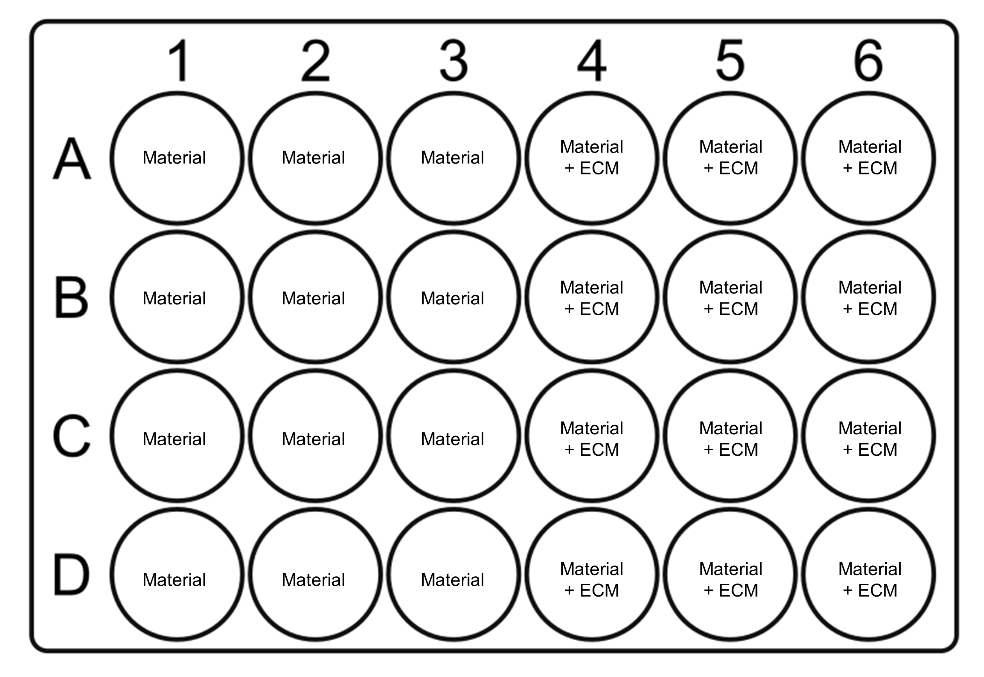


**Fig. S2** 24-well plate arrangement for testing of different chip materials (PDMS or thiol-ene) on steroid adsorption. ECM: extracellular matrix, referring to coating agent collagen IV.


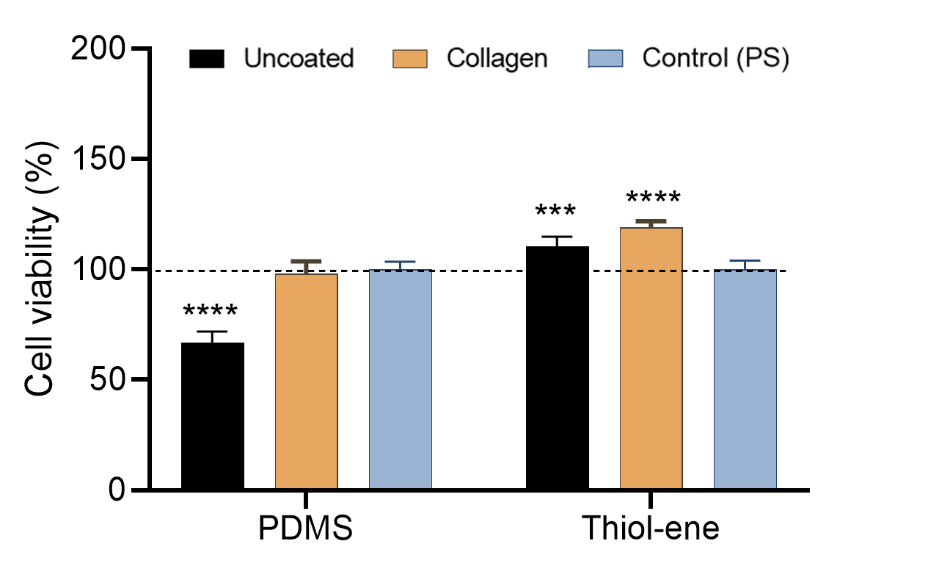


**Fig. S3** H295R cell viability 72 h after seeding (y-axis) on PDMS and thiol-ene discs compared to polystyrene control (PS =100%) with and without collagen coating (x-axis). Values represent mean ± SD (n = 5-6) and asterisks indicate significant difference from control (one-way ANOVA, post hoc Dunnett’s test). Significance levels: *: p ≤ 0.05, **: p ≤ 0.01, ***: p ≤ 0.001 and ****: p ≤ 0.0001


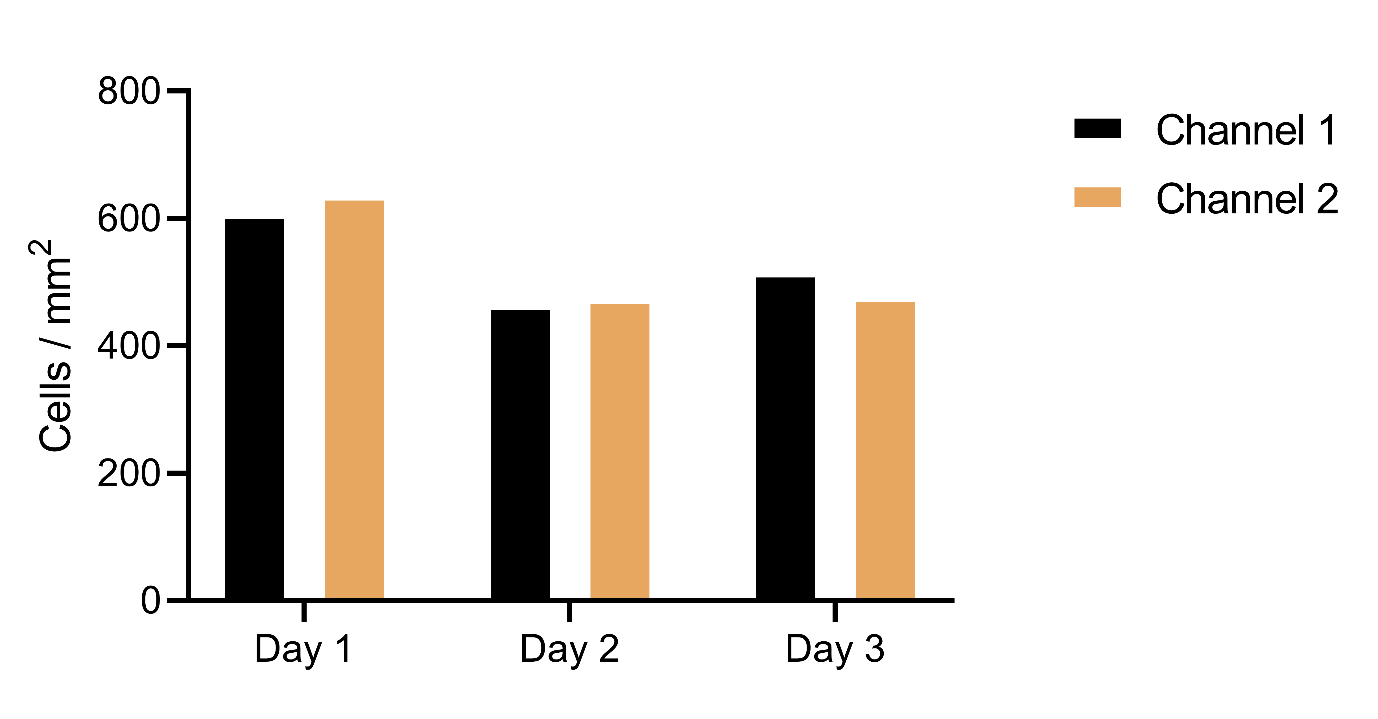


**Fig. S4** Mean cell count/mm^2^ on chip 2 h after seeding (x-axis) of H295R cell suspensions prepared on 3 different days (y-axis). Cell seeding was carried out in duplicates (channel 1 and 2). Cell count is the average of 9 positions counted in each channel.


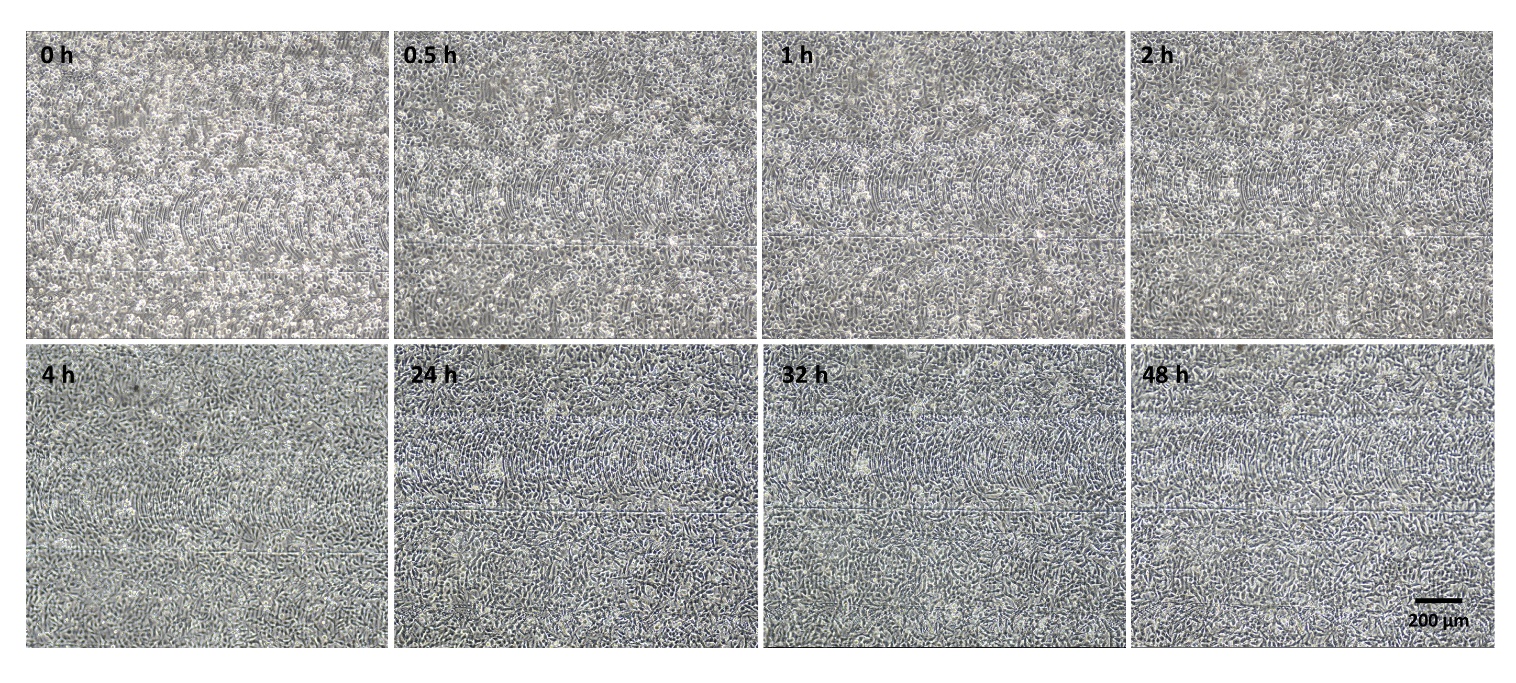


**Fig. S5** H295R cell attachment over 48 h in microfluidic thiol-ene channel with 0.01 mg/mL collagen coating. The top row shows cell settling 0-2h after seeding without flow. The bottom row depicts cell morphology 2 to 48 h after initiation of 10 µL/min flow.


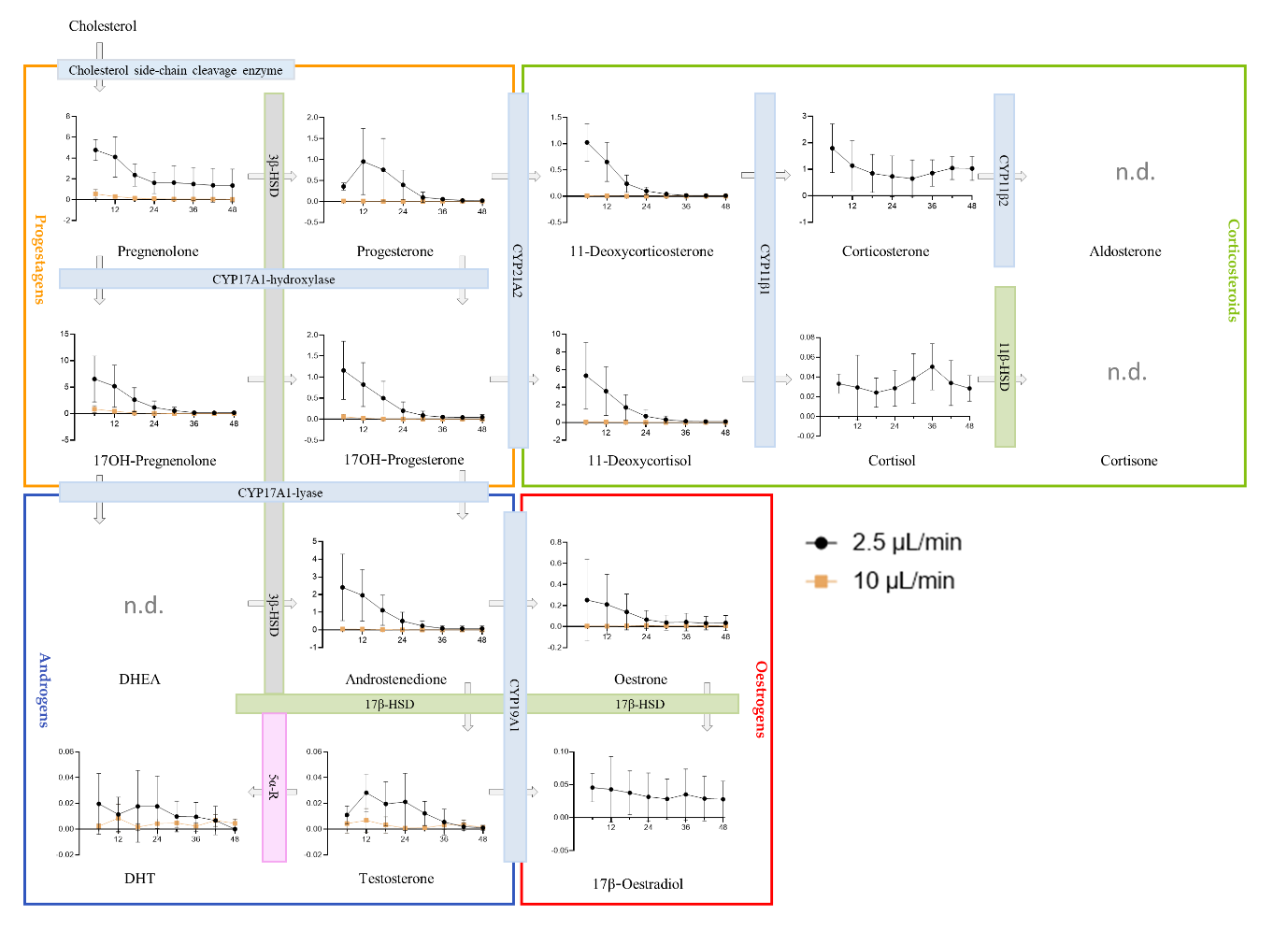


**Fig. S6** Mean H295R steroid production on thiol-ene chip (ng/mL; y-axis), collected in 6h fractions over 48 h (x-axis). Values represent mean ± SD (n = 3-4). n.d.: not determined. Blue bars: Cytochrome P-540 enzymes, Grey and green bars: Hydroxysteroid dehydrogenase enzymes. Pink bar: 5α-reductase.

## Supplementary material tables

**Table S1** H295R cell viability 72 h after seeding, on PDMS and thiol-ene discs and polystyrene control (PS =100%) with and without collagen coating. Values represent mean ± SD (n = 5-6)

|  | **PDMS** |  | | **Thiol-ene** |
| --- | --- | --- | --- | --- |
| Uncoated | 66.9 ± 5.0 % | | 114.6 ± 2.6 % | |
| Collagen | 98.0 ± 5.8 % | | 120.4 ± 3.3 % | |
| Unpaired t-test | p<0.0001 | | p<0.01 | |
|  |  | |  | |
| Control (PS) | 100.0 ± 3.6 % | | 100.0 ± 4.2 % | |
|  | | | | |

**Table S2** H295R steroid hormone levels in cell medium collected after 24 h incubation on PDMS and thiol-ene discs, with and without collagen coating. Steroid levels are normalized to polystyrene control (PS = 100.0 %). Values represent mean ± SD (n = 5-6). NA: not applicable; ^a^not detected; ^b^not included.

| **Steroid** | **PDMS** | | |  | **Thiol-ene** | | |  | **PS (Control)** |
| --- | --- | --- | --- | --- | --- | --- | --- | --- | --- |
|  | Uncoated |  | Collagen |  | Uncoated |  | Collagen |  |  |
| ***Progestagens*** |  |  |  |  |  |  |  |  |  |
| Pregnenolone | 68.3 ± 19.9% |  | 98.8 ± 26.1% |  | 123.5 ± 41.3% |  | 121.8 ± 26.6% |  | 100.0 ± 26.8% |
| 17-Hydroxypregenenolone | 70.2 ± 9.4% |  | 66.9 ± 10.4% |  | 115.3 ± 16.7% |  | 111.5 ± 30.0% |  | 100.0 ± 24.7% |
| Progesterone | 62.0 ± 8.4% |  | 50.8 ± 8.1% |  | 129 ± 14.5% |  | 117.1 ± 25.7% |  | 100.0 ± 6.2% |
| 17-Hydroxyprogesterone | 73.3 ± 12.4% |  | 64.5 ± 16.3% |  | 127.1 ± 14.3% |  | 121.0 ± 36.8% |  | 100.0 ± 21.8% |
| ***Corticosteroids*** |  |  |  |  |  |  |  |  |  |
| Cortisol | 75.7 ± 19.4% |  | 47.7 ± 33.6% |  | 56.4 ± 24.9% |  | 58.1 ± 27.1% |  | 100.0 ± 52.6% |
| 11-Deoxycortisol | 60.6 ± 9.4% |  | 47.3 ± 4.0% |  | 104.4 ± 9.0% |  | 107.0 ± 20.9% |  | 100.2 ± 11.4% |
| Corticosterone | 53.9 ± 3.9% |  | 49.2 ± 3.2% |  | 102.8 ± 19.2% |  | 107.6 ± 22.0% |  | 100.0 ± 15.7% |
| 11-Deoxycorticosterone | 23.7 ± 3.5% |  | 16.6 ± 0.7% |  | 113.7 ± 7.6% |  | 109.8 ± 18.9% |  | 100.0 ± 13.1% |
| Cortisone | 50.6 ± 4.7% |  | 63.4 ± 27.2% |  | 92.9 ± 25.1% |  | 105.9 ± 44.6% |  | 100.0 ± 37.6% |
| Aldosterone | NA^b^ |  | NA^b^ |  | NA^b^ |  | NA^b^ |  | NA^b^ |
| ***Androgens*** |  |  |  |  |  |  |  |  |  |
| Androstenedione | 17.2 ± 1.9% |  | 16.0 ± 1.6% |  | 101.5 ± 2.5% |  | 101.9 ± 22.1% |  | 100.0 ± 8.4% |
| Dehydroepiandrosterone | 62.5 ± 4.8% |  | 73.4 ± 10.8% |  | 102.8 ± 19.1% |  | 105.5 ± 24.9% |  | 100.0 ± 20.9% |
| Testosterone | 37.8 ± 11.3% |  | 36.7 ± 7.8% |  | 108.2 ± 13.3% |  | 101.5 ± 20.1% |  | 100.0 ± 8.7% |
| Dihydrotestosterone | NA^b^ |  | NA^b^ |  | NA^b^ |  | NA^b^ |  | NA^b^ |
| ***Estrogens*** |  |  |  |  |  |  |  |  |  |
| Estrone | 42.9 ± 4.2% |  | 35.6 ± 5% |  | 112.5 ± 8.6% |  | 112.3 ± 26.9% |  | 100.0 ± 8.6% |
| 17β-Estradiol | 76.3 ± 5.8% |  | 75.9 ± 13.3% |  | 109.4 ± 11.0% |  | 120.5 ± 23.0% |  | 100.0 ± 7.0% |

**Table S3** Steroid hormone recovery of 1 ng/mL spiked cell medium collected after 24 h incubation on PDMS and thiol-ene discs in a 24 well-plate. Uncoated PS wells served as control. Hormone levels are expressed as percentage of reference solution (1 ng/mL steroid mix in cell medium). Values represent mean ± SD (n = 6-12). NA: not applicable; ^a^not detected; ^b^not included.

| **Steroid** | **PDMS** | | |  | **Thiol-ene** | | |  | **PS (Control)** |
| --- | --- | --- | --- | --- | --- | --- | --- | --- | --- |
|  | Uncoated |  | Collagen |  | Uncoated |  | Collagen |  |  |
| ***Progestagens*** |  |  |  |  |  |  |  |  |  |
| Pregnenolone | 14.2 ± 5.7% |  | 23.3 ± 17.9% |  | 50.2 ± 3.5% |  | 50.8 ± 3.6% |  | 67.7 ± 10.8% |
| 17-Hydroxypregenenolone | 51.4 ± 7.7% |  | 49.0 ± 5.3% |  | 92.7 ± 8.4% |  | 92.1 ± 5.5% |  | 108.3 ± 23% |
| Progesterone | 1.4 ± 0.3% |  | 1.2 ± 0.3% |  | 125.4 ± 16.2% |  | 121.6 ± 16.5% |  | 77.0 ± 6.3% |
| 17-Hydroxyprogesterone | 12.7 ± 2.2% |  | 11.6 ± 1.2% |  | 83.3 ± 5.4% |  | 82.4 ± 2.5% |  | 101.3 ± 13.0% |
| ***Corticosteroids*** |  |  |  |  |  |  |  |  |  |
| Cortisol | 84.3 ± 5.9% |  | 81.6 ± 5.2% |  | 93.6 ± 4.4% |  | 93.2 ± 5.1% |  | 84.8 ± 4.7% |
| 11-Deoxycortisol | 88.1 ± 7.7% |  | 91.7 ± 7.9% |  | 93.9 ± 2.9% |  | 91.4 ± 4.6% |  | 76.7 ± 7.5% |
| Corticosterone | 94.3 ± 6.6% |  | 89.4 ± 7.8% |  | 80.5 ± 4.6% |  | 76.5 ± 5.7% |  | 85.7 ± 6.7% |
| 11-Deoxycorticosterone | 13.2 ± 2% |  | 13.1 ± 2.2% |  | 74.6 ± 3.7% |  | 72.4 ± 5.5% |  | 84.3 ± 7.1% |
| Cortisone | 94.9 ± 6.2% |  | 95.2 ± 6.0% |  | 87.0 ± 5.9% |  | 87.2 ± 8.0% |  | 82.8 ± 7.7% |
| Aldosterone | NA^b^ |  | NA^b^ |  | NA^b^ |  | NA^b^ |  | NA^b^ |
| ***Androgens*** |  |  |  |  |  |  |  |  |  |
| Androstenedione | 3.6 ± 1.1% |  | 4.2 ± 2.7% |  | 90.0 ± 2.1% |  | 90.9 ± 4.6% |  | 93.8 ± 8.2% |
| Dehydroepiandrosterone | 22.8 ± 2.6% |  | 23.2 ± 2.6% |  | 91.3 ± 6.4% |  | 90.5 ± 1.5% |  | 85.4 ± 12.9% |
| Testosterone | 16.0 ± 2.0% |  | 14.3 ± 3.5% |  | 85.4 ± 2.9% |  | 86.3 ± 4.0% |  | 90.0 ± 6.6% |
| Dihydrotestosterone | NA^b^ |  | NA^b^ |  | NA^b^ |  | NA^b^ |  | NA^b^ |
| ***Estrogens*** |  |  |  |  |  |  |  |  |  |
| Estrone | 41.0 ± 4.9% |  | 38.4 ± 6.2% |  | 87.3 ± 5.3% |  | 85.9 ± 4.2% |  | 88.9 ± 17.2% |
| 17β-Estradiol | 63.9 ± 8.1% |  | 65.2 ± 4.3% |  | 63.8 ± 13.1% |  | 65.2 ± 13.4% |  | 81.0 ± 13.3% |

**Table S4** Steroid recovery of 5 ng/mL collected over 48 h at a 10 µL/min flowrate. Recoveries for integral microfluidic set- up (collagen coated thiol-ene chip, plastic syringe and PEEK tubing) and auxiliary components (syringe and PEEK tubing) are expressed as percentage of control (5 ng/mL steroid mix in cell medium). Values represent mean ± SD (n = 3-6). NA: not applicable; ^a^not detected; ^b^not included.

| **Steroid** | **Integral microfluidic setup**  **(On-chip recovery)** | | |  | | **Syringe + tubing**  **(Reference)** | | |  | **Control** | | |
| --- | --- | --- | --- | --- | --- | --- | --- | --- | --- | --- | --- | --- |
|  | 0 – 24 h |  | 24 – 48 h |  | 0 – 24 h | |  | 24 – 48 h |  | 0 – 24 h |  | 24 – 48 h |
| ***Progestagens*** |  |  |  |  |  | |  |  |  |  |  |  |
| Pregnenolone | 69.1 ± 18.0% |  | 37.0 ± 9.9% |  | 80.6 ± 24.2% | |  | 21.6 ± 3.8% |  | 100.0 ± 5.1% |  | 100.0 ± 4.7% |
| 17-Hydroxypregenenolone | 98.9 ± 13.9% |  | 87.4 ± 14.7% |  | 99.5 ± 5.5% | |  | 86.5 ± 8.3% |  | 100.0 ± 3.7% |  | 100.0 ± 5.5% |
| Progesterone | 51.5 ± 25.3% |  | 15.5 ± 5.4% |  | 70.2 ± 26% | |  | 7.7 ± 1.9% |  | 100.0 ± 3.5% |  | 100.0 ± 5.7% |
| 17-Hydroxyprogesterone | 88.9 ± 25.4% |  | 63.1 ± 22.9% |  | 94.0 ± 13.4% | |  | 57.5 ± 7.9% |  | 100.0 ± 3.3% |  | 100.0 ± 2.4% |
| ***Corticosteroids*** |  |  |  |  |  | |  |  |  |  |  |  |
| Cortisol | 99.0 ± 14.4% |  | 97.3 ± 13.7% |  | 100.0 ± 9.4% | |  | 93.1 ± 13.3% |  | 100.0 ± 10.4% |  | 100.0 ± 8.6% |
| 11-Deoxycortisol | 95.2 ± 14.5% |  | 82.3 ± 14.1% |  | 96.0 ± 9.5% | |  | 80.0 ± 6.4% |  | 100.0 ± 7.9% |  | 100.0 ± 5.6% |
| Corticosterone | 101.5 ± 10.6% |  | 97.9 ± 10.0% |  | 97.5 ± 7.7% | |  | 94.1 ± 8.3% |  | 100.0 ± 7.1% |  | 100.0 ± 6.0% |
| 11-Deoxycorticosterone | 72.0 ± 21.2% |  | 40.9 ± 10.0% |  | 81.3 ± 23.8% | |  | 27.3 ± 5.1% |  | 100.0 ± 5.1% |  | 100.0 ± 5.7% |
| Cortisone | 95.8 ± 11.9% |  | 95.7 ± 13.2% |  | 92.1 ± 11.0% | |  | 91.4 ± 13.3% |  | 100.0 ± 10.7% |  | 100.0 ± 11.3% |
| Aldosterone | 100.9 ± 7.3% |  | 100.0 ± 9.5% |  | 97.3 ± 6.7% | |  | 96.8 ± 6.8% |  | 100.0 ± 2.4% |  | 100.0 ± 4.3% |
| ***Androgens*** |  |  |  |  |  | |  |  |  |  |  |  |
| Androstenedione | 76.5 ± 17.7% |  | 50.4 ± 10.7% |  | 80.2 ± 21.6% | |  | 30.0 ± 7.3% |  | 100.0 ± 5.3% |  | 100.0 ± 5.4% |
| Dehydroepiandrosterone | 88.6 ± 16.7% |  | 67.6 ± 14.1% |  | 90.9 ± 17.3% | |  | 50.0± 8.1% |  | 100.0 ± 7.7% |  | 100.0 ± 7.4% |
| Testosterone | 87.4 ± 14.6% |  | 69.6 ± 12.5% |  | 87.3 ± 14.5% | |  | 52.8 ± 8.4% |  | 100.0 ± 6.6% |  | 100.0 ± 6.4% |
| Dihydrotestosterone | 80.1 ± 16.9% |  | 58.1 ± 10.4% |  | 85.7 ± 16.3% | |  | 49.0 ± 5.8% |  | 100.0 ± 5.3% |  | 100.0 ± 6.0% |
| ***Estrogens*** |  |  |  |  |  | |  |  |  |  |  |  |
| Estrone | 82.8 ± 13.9% |  | 67.8 ± 10.8% |  | 91.0 ± 14.7% | |  | 53.3 ± 9.2% |  | 100.0 ± 4.8% |  | 100.0 ± 5.6% |
| 17β-Estradiol | 91.7 ± 13.8% |  | 82.5 ± 12.1% |  | 96.0 ± 8.7% | |  | 75.1 ± 8.2% |  | 100.0 ± 5.3% |  | 100.0 ± 6.1% |

**Table S5** Cell seeding uniformity, illustrated by cell distribution per position (1-9) in microfluidic thiol-ene channel. Values are expressed as average cell count per mm^2^ ± relative standard deviation (RSD), and as relative deviation from mean cell count per channel (%). (n =6)

| ***Position*** | ***Avg. cells/mm^2^ ± RSD*** *(n=6)* | | ***Rel. dev. from mean (%)*** |  | ***Position*** | | ***Avg. cells/mm^2^ ± RSD*** *(n=18)* | ***Rel. dev. from mean (%)*** | | **Uniformity**  (One way ANOVA) |
| --- | --- | --- | --- | --- | --- | --- | --- | --- | --- | --- |
|  |  | |  |  |  | |  |  |  | |
| 1 | 529.6 ± 22.0% | | +0.5% |  | **Horizontal** | |  |  |  | |
| 2 | 499.9 ± 22.1% | | -5.2% |  |  |  |  |  |  | |
| 3 | 549.0 ± 17.4% | | +4.2% |  | 1, 2, 3 | Front | 526.2 ± 19.6% | -0.2% | p = 0.6998 | |
| 4 | 523.3 ± 24.2% | | -0.7% |  | 4, 5, 6 | Middle | 517.7 ± 17.7% | -1.8% |  |  |
| 5 | 501.5 ± 12.9% | | -4.8% |  | 7, 8, 9 | End | 537.3 ± 19.6% | 2.0% |  |  |
| 6 | 528.2 ± 16.8% | | +0.2% |  |  | |  |  |  | |
| 7 | 553.1 ± 22.0% | | +4.9% |  | ***Mean ± RSD*** | | 527.0 ± 1.9% |  |  | |
| 8 | 544.3 ± 17.8% | | +3.3% |  |  | |  |  |  | |
| 9 | 514.4 ± 21.7% | | -2.4% |  | **Vertical** | |  |  |  | |
|  |  | |  |  |  |  |  |  |  | |
|  |  | |  |  | 1, 4, 7 | Top | 535.3 ± 21.5% | 0.9% | p = 0.8003 | |
| ***Mean ± RSD*** | | 527.0 ± 22.4% | |  | 2, 5, 8 | Middle | 515.2 ± 17.5% | -1.8% |  |  |
|  |  | | |  | 3, 6, 9 | Bottom | 530.6 ± 17.7% | 0.8% |  |  |
| **Uniformity**  (One way ANOVA) | | | p = 0.8935 |  |  | |  |  |  | |
|  |  |  |  |  | ***Mean ± RSD*** | | 527.0 ± 2.0% |  |  | |

**Table S6** Cell seeding repeatability, illustrated by average cell counts from cell suspension seeded in duplicates (channel 1 & 2) on 3 different days. The average cells/mm^2^ was calculated from 9 positions counted in each channel.

|  | ***Channel*** | ***Avg. cells/mm^2^*** | ***Avg. cells/channel (72 mm^2^)*** |
| --- | --- | --- | --- |
| ***Day 1*** | 1 | 605.4 | 4.5 × 10^4^ |
|  | 2 | 634.6 |  |
| ***Day 2*** | 1 | 462.3 | 3.4 × 10^4^ |
|  | 2 | 471.6 |  |
| ***Day 3*** | 1 | 513.5 | 3.6 × 10^4^ |
|  | 2 | 474.7 |  |
| ***Mean ± RSD*** | |  | 3.8 × 10^4^ ± 15.5 % |
| **Two-way ANOVA** | | **Intra –day variation** | **Inter –day variation** |
|  |  | ns | p < 0.05 |

### Spike-recovery experiment from microfluidic set-up

For time-resolved assessment of steroid recovery from the entire microfluidic setup, the microfluidic chip was prepared as follows: One day before cell seeding, the microchip was disinfected with 70% ethanol and rinsed with sterile PBS, each for 15 minutes and at a flow rate of 20 µL/min. Subsequently, 0.01 mg/mL collagen IV was introduced to coat the chip for cell attachment and left in the cell culture incubator overnight. To remove the extra collagen before use, the chip was rinsed with cell medium for 20 minutes at a flow rate of 10 µL/min. To determine steroid recovery from the microfluidic set-up (including plastic syringe, thiol-ene chip and PEEK tubing), a 1 ng/mL steroid mix in cell medium was run through 2 channels simultaneously and collected into 16 x 3 h fractions, over 48 h. As a reference, steroid mix was collected into 3 h fractions in Eppendorf tubes over 48 h, directly from a PEEK line connected to a syringe (without going through the chip). 5 ng/mL steroid mix in Eppendorf tubes served as control samples for each time point and were left open for the same time duration with lid open in sample collector, to account for evaporation effects. The experiment was conducted 3 times in total on 3 different days. Samples were stored at -20 ℃ until sample clean-up. For data analysis, recoveries from the on-chip recovery and reference set were expressed as percentage of control.

Steroid recoveries from the integral microfluidic system as well as auxiliary components (plastic syringe and PEEK microtubing feeding cell medium to the chip and into Eppendorf tubes) are depicted in Table S4 for time intervals 0-24h and 24-48h. For relatively hydrophilic corticosteroids (COS, ALDO, COR, CORNE) recovered steroid levels remained constant over time, while a sharp drop was observed for rather hydrophobic structures such as PREG, PROG, AN, 11 deoxy-COS. Notably in the 24-48h timeframe, recoveries are consistently lower in the reference set, indicating that auxiliary microfluidic equipment likely retains a great proportion of steroids, rather than the thiol-ene chip itself, and that adsorption increases with time. Including a reference set was relevant to this recovery experiment, as steroid spiked reference solution was injected via a plastic syringe. However, it should be noted that in a cell-based steroidogenesis assay, the syringe would solely serve the purpose of infusing the chip with cell medium and would not come into contact with steroids secreted by cells. Syringe steroid adsorption is therefore negligible for our microfluidic system.

**Table S7** Lowest to highest measured steroid concentrations (ng/mL) produced by H295R cells on thiol-ene chip. (n=3-4). NA: not applicable; ^a^not detected; ^b^not included. NA: not applicable; ^a^not detected; ^b^not included.

|  | **2.5 µL/min** | | |  | **10 µL/min** | | |  | **LLOD** | **LLOQ** |
| --- | --- | --- | --- | --- | --- | --- | --- | --- | --- | --- |
|  | 0- 24 h |  | 24- 48 h |  | 0- 24 h |  | 24- 48 h |  |  |  |
| ***Progestagens*** |  |  |  |  |  |  |  |  |  |  |
| Pregnenolone | 0.08 - 1.92 |  | 0 - 0.28 |  | 0 - 0.03 |  | 0 - 0.03 |  | 0.0589 | 0.1784 |
| 17-Hydroxypregenenolone | 0.30 - 11.6 |  | 0 - 1.49 |  | 0 - 1.6 |  | 0 - 0.05 |  | 0.1231 | 0.3730 |
| Progesterone | 0.08 - 1.92 |  | 0 - 0.28 |  | 0 - 0.03 |  | 0 - 0.03 |  | 0.0176 | 0.0334 |
| 17-Hydroxyprogesterone | 0 - 1.79 |  | 0 - 0.21 |  | 0 - 0.14 |  | NA^a^ |  | 0.1888 | 0.5725 |
| ***Corticosteroids*** |  |  |  |  |  |  |  |  |  |  |
| Cortisol | 0.01 - 0.07 |  | 0.01 - 0.07 |  | 0 - 0.04 |  | 0 - 0.11 |  | 0.0301 | 0.0911 |
| 11-Deoxycortisol | 0.05 - 8.93 |  | 0 - 0.90 |  | 0 - 0.1 |  | 0 - 0.04 |  | 0.0358 | 0.1086 |
| Corticosterone | 0.19 - 2.76 |  | 0.04 - 1.61 |  | NA^a^ |  | NA^a^ |  | 0.3634 | 1.1012 |
| 11-Deoxycorticosterone | 0 - 1.43 |  | 0 - 0.09 |  | 0 - 0.02 |  | 0 - 0.01 |  | 0.0117 | 0.0355 |
| Cortisone | NA^b^ |  | NA^b^ |  | NA^b^ |  | NA^b^ |  | 0.0210 | 0.0637 |
| Aldosterone | NA^b^ |  | NA^b^ |  | NA^b^ |  | NA^b^ |  | 0.0258 | 0.0781 |
| ***Androgens*** |  |  |  |  |  |  |  |  |  |  |
| Androstenedione | 0.08 - 4.76 |  | 0 - 0.60 |  | 0 - 0.09 |  | 0 - 0.01 |  | 0.0217 | 0.0657 |
| Dehydroepiandrosterone | NA^b^ |  | NA^b^ |  | NA^b^ |  | NA^b^ |  | 0.1384 | 0.4193 |
| Testosterone | 0 - 0.05 |  | 0 - 0.02 |  | 0 - 0.02 |  | 0 - 0.01 |  | 0.0334 | 0.1013 |
| Dihydrotestosterone | 0 – 0.06 |  | 0 – 0.02 |  | 0 – 0.02 |  | 0 – 0.01 |  | 0.0849 | 0.2571 |
| ***Estrogens*** |  |  |  |  |  |  |  |  |  |  |
| Estrone | 0 - 0.82 |  | 0 - 0.17 |  | 0 - 0.02 |  | 0 - 0.01 |  | 0.0547 | 0.1658 |
| 17β-Estradiol | 0 - 0.09 |  | 0 - 0.07 |  | NA^a^ |  | NA^a^ |  | 0.0262 | 0.0794 |
